# Innovative vibriosis control in open aquaculture: *Paratapes undulata* as a sustainable growth and resistance enhancer in red tilapia

**DOI:** 10.1038/s41598-025-01026-x

**Published:** 2025-05-22

**Authors:** Rehab E. Mowafy, Hend M. Megahed, Sally H. Abou Khadra, Mohamed A. Bakry, Ahmed Hussien Moustafa, Azza SalahEldin El-Demerdash

**Affiliations:** 1 Department of Pathology, Agricultural Research Center (ARC), Animal Health Research Institute (AHRI), Zagazig, 44516 Egypt; 2 Department of Biochemistry, Agricultural Research Center (ARC), Animal Health Research Institute (AHRI), Zagazig, 44516 Egypt; 3 Department of Microbiology, Agricultural Research Center (ARC), Animal Health Research Institute (AHRI), Zagazig, 44516 Egypt; 4 Department of Fish Health, Agricultural Research Center (ARC), Animal Health Research Institute (AHRI), Zagazig, 44516 Egypt; 5 Department of Chemistry, Faculty of Science, Zagazig University, Zagazig, 44519 Egypt; 6 Laboratory of Biotechnology, Department of Microbiology, Agricultural Research Center (ARC), Animal Health Research Institute (AHRI), Zagazig, 44516 Egypt

**Keywords:** Aquaculture immunology, Natural antimicrobials, *Paratapes undulata*, Probiotic aquaculture, *Vibrio alginolyticus*, Immunology, Microbiology, Molecular biology, Biomarkers

## Abstract

This study demonstrates the novel therapeutic potential of *Paratapes undulata* for mitigating *Vibrio alginolyticus* infection in red tilapia. In vivo, *P. undulata* significantly improved growth by approximately 362% in group G3 (Clam-treated Control) and 284% in group G4 (Clam-treated Infected), compared to the infected control group (G2), and reduced mortality by 100% in group G3 and 75% in group G4, compared to the infected control group (G2), and alleviated clinical signs, correlating with enhanced hematological and biochemical profiles, and reduced tissue damage. Mechanistically, *P. undulata* modulated the immune response by shifting cytokine balance towards anti-inflammation, enhanced antioxidant capacity, and directly inhibited *Vibrio alginolyticus* virulence. Gas Chromatography–Mass Spectrometry and Fourier-Transform Infrared Spectroscopy analyses revealed the presence of bioactive compounds contributing to these effects. These findings establish *P. undulata* as a promising, natural, and sustainable biocontrol agent for vibriosis in aquaculture, offering a novel strategy for disease management and reducing reliance on antibiotics. This study suggests that *P. undulata* can be effectively incorporated into aquaculture feed or water treatments to prevent and manage vibriosis outbreaks.

## Introduction

The global aquaculture industry, crucial for sustainable food production, faces significant challenges from bacterial pathogens like *Vibrio alginolyticus*, which causes substantial economic losses and environmental concerns^1^. Red tilapia (*Oreochromis* sp.), a commercially important species in aquaculture, is particularly susceptible to *V. alginolyticus* infections, often cultured in open systems that heighten vulnerability^2,3^. This susceptibility, coupled with its status as a well-established model for aquaculture disease studies, underscores the urgent need for effective and sustainable control strategies. *V. alginolyticus* was selected due to its recognized prevalence and virulence in red tilapia aquaculture, and its status as a major cause of vibriosis in these open culture systems^4,5^. Specifically, studies show that red tilapia exhibits a high susceptibility to *V. alginolyticus* compared to other *Vibrio* species, resulting in significant mortality and morbidity^6,7^. Furthermore, *V. alginolyticus* is frequently isolated from diseased red tilapia in various geographical regions, indicating its widespread impact^2,6,8^. These factors, in addition to its zoonotic potential, make *V. alginolyticus* a particularly relevant and impactful pathogen to study in the context of red tilapia aquaculture^9,10^.

This study pioneers the use of *Paratapes undulata*, a locally abundant bivalve, as a novel biocontrol agent against *V. alginolyticus*. *P. undulata* was selected due to its unique profile of bioactive compounds, including terpenoids, alkaloids, and polyphenols, suggesting a robust natural defense mechanism^11,12^. Unlike other marine bivalves, *P. undulata* demonstrates a particularly high accumulation of these compounds, and its prevalence in aquaculture environments experiencing *V. alginolyticus* outbreaks implies potential ecological interactions. While other natural antimicrobial agents exist, *P. undulata’s* broad-spectrum activity, high compound stability, and ease of access make it a promising candidate for sustainable vibriosis control^13–15^. Previous studies have documented the antimicrobial, antioxidant, and immunomodulatory properties of bioactive compounds in marine bivalves^16–18^. *P. undulata* accumulates diverse secondary metabolites, such as terpenoids, alkaloids, and polyphenols, known for these biological activities.

Previous studies have explored the potential of various marine-derived compounds for vibriosis control, including extracts from marine algae and other bivalve species^19,20^. These studies have demonstrated promising antimicrobial activities; however, they often report limitations such as low compound stability, limited availability, or narrow-spectrum activity. In contrast, *P. undulata* presents a unique advantage due to its high accumulation of diverse bioactive compounds, its broad-spectrum antimicrobial activity, and its local abundance. Furthermore, unlike previous studies that primarily focused on antimicrobial effects, this research provides a comprehensive analysis of the immunomodulatory effects of *P. undulata* and identifies the specific bioactive compounds responsible for its therapeutic potential. This study is the first to investigate the use of *P. undulata* as a biocontrol agent against *V. alginolyticus* in red tilapia, offering novel insights into sustainable vibriosis management.

By mimicking natural ecological interactions and exploring the bioactive potential of *P. undulata*, this research aims to identify and characterize novel compounds with potent antimicrobial activity. This work contributes to the development of sustainable and eco-friendly disease prevention and control strategies in aquaculture, minimizing reliance on antibiotics and promoting aquatic ecosystem health.

## Materials and methods

### Ethical approval

This study and all methods were approved by the Institutional Animal Care and Use Committee (IACUC) of the Faculty of Veterinary Medicine, Zagazig University, Egypt (Approval number: ZU-IACUC/2/F/343/2023) and adhered to the ARRIVE guidelines (PLoS Bio 8(6), e1000412,2010). All experiments were performed in accordance with relevant guidelines and regulations.

### Sample collection and bacterial isolation

A total of 100 red tilapia fish were collected from local fish markets and immediately transported to the laboratory on ice to minimize bacterial degradation. Sterile techniques were employed to collect samples from the internal organs (liver, kidney, gills, and spleen) of each fish. The organs were homogenized and inoculated into 1% peptone water containing 3% NaCl. The inoculated media were then incubated aerobically at 37 °C for 18–24 h. Subsequently, inocula were streaked onto Thiosulfate Citrate Bile Sucrose (TCBS) agar plates (Oxoid, UK) and incubated at 37 °C for 24 h. TCBS agar is a selective and differential medium specifically designed for the isolation of *Vibrio* species, including *V. alginolyticus*. It contains bile salts and a high pH, which inhibit the growth of most other bacteria while allowing *Vibrio* species to thrive^21,22^. Colonies with characteristic yellow, smooth morphology were counted to determine the Vibrio count. Gram staining and a series of biochemical tests, including oxidase, catalase, indole production, citrate utilization, urease, triple sugar iron, gelatin hydrolysis, and methyl red tests, were performed to confirm the identity of the isolates as *V. alginolyticus*^23–25^.

### Antibiotic susceptibility testing

The antimicrobial susceptibility of the *V. alginolyticus* isolates was determined using the Kirby-Bauer disk diffusion method^26^. The following antimicrobial agents (Oxoid, UK) were tested: ampicillin (AM/10μg), erythromycin (E/15μg), oxolinic acid (OA/2μg), oxytetracycline (OT/30μg), doxycycline (DO/30μg), florfenicol (FFC/30μg), and trimethoprim/sulfamethoxazole (SXT/25μg). Results were interpreted according to CLSI^27^ guidelines. For this analysis, multiple *V. alginolyticus* isolates were tested, and the susceptibility results were compiled to determine the overall resistance profile. The multiple antimicrobial resistance indices (MARI) were calculated as previously described by Tambekar et al.^28^. These indices were helpful to categorize isolates based on their resistance profiles, including:

- **Pan-drug resistant (PDR):** Defined by CLSI as non-susceptibility to all agents in all antimicrobial categories tested.
- **Extensively drug-resistant (XDR):** Defined by CLSI as non-susceptibility to at least one agent in all but two or fewer antimicrobial categories (i.e., bacterial isolates remain susceptible to only one or two categories).

### Molecular identification and genomic characterization of *V. alginolyticus* isolates

Presumptive *V. alginolyticus* colonies were grown overnight in Tryptone Soy Broth (TSB; Oxoid, USA). Genomic DNA was extracted using a QIAamp DNA Mini kit (Qiagen GmbH, Hilden, Germany).

#### Virulence gene PCR

Polymerase chain reaction (PCR) amplification was performed using oligonucleotide primers targeting *Vibrio* species’ *tdh*, *toxR*, and *trh* genes (Table 1). PCR conditions were: initial denaturation at 95 °C for 5 min, 35 cycles of 95 °C for 30 s, annealing at T°C, 72 °C for 1 min, and a final extension at 72 °C for 7 min. The reaction mixture contained 5 μL template DNA, 1 μL of each primer (10 μM), 12.5 μL 2 × DreamTaq PCR Master Mix (Qiagen), and 9.5 μL sterile water. *Vibrio* species were initially identified by these virulence gene PCRs. Specific identification of *V. alginolyticus* was achieved by combining these results with phenotypic and biochemical characterization. The combination of positive virulence gene PCRs and *V. alginolyticus*-consistent phenotypic/biochemical profiles confirmed the isolates’ identity. *V. alginolyticus* strain ATCC® 17,749™ (American Type Culture Collection, Manassas, VA, USA) served as a positive control (https://thermofisher.com/microbiology). A mixture without DNA template as negative control was included in each PCR run.

**Table 1:** Primers sequences and cycling conditions used for molecular identification and virulence quantification of vibrio.

Target gene	Primers sequences	Amplicon size (bp)	Annealing (°C)	Reference
*16S rRNA*	F; GGCGTAAAGCGCATGCAGGT R; GAAATTCTACCCCCCTCTACAG	114	50	^29^
*Tdh*	F; GTAAAGGTCTCTGACTTTTGGAC R; TGGAATATGAACCTTCATCTTCACC	270	60	^30^
*toxR*	F; GAC GCA ATC GTT GAA CCA GAA R; GCA AAT CGG TAG TAA TAG TGC CAA	117	60	^31^
*Trh*	F; TTCACAAAATCAGAAAAAACAAGA R; TTTAATTTTGTGACATACATTCATC	217	60	^30^
*V. alginolyticus* (*collagenase*)	F; CGAGTACAGTCACTTGAAAGCC R; CACAACAGAACTCGCGTTACC	738	57	^32^

#### Collagenase gene PCR

Molecular identification using collagenase gene PCR (738 bp band) further confirmed the isolates’ identity. PCR conditions were: initial denaturation at 95 °C for 5 min, 35 cycles of 95 °C for 30 s, annealing at 57 °C, 72 °C for 1 min, and a final extension at 72 °C for 7 min. The reaction mixture contained 5 μL template DNA, 1 μL of each primer (10 μM), 12.5 μL 2 × DreamTaq PCR Master Mix (Qiagen), and 4.5 μL sterile water.

### Identification of the challenged *V. alginolyticus* isolate based on 16S rRNA gene sequencing

To confirm the identity of the challenged strain, which was previously isolated from the same aquaculture environment, using a robust and reliable method, partial 16S rRNA gene sequencing was chosen as the most stringent confirmatory method for this purpose. While we initially employed phenotypic, biochemical tests, and PCR with *Vibrio* virulence genes for preliminary identification, 16S rRNA sequencing provided the necessary definitive identification of the challenged strain.

The rationale behind this approach was to ensure the highest level of confidence in the identity of the pathogen used for the challenge. By using 16S rRNA sequencing, we could directly compare the sequence of our isolate to established databases and confirm its identity as *V. alginolyticus*. This was crucial for ensuring the reproducibility and validity of our experimental results.

A single *V. alginolyticus* isolate exhibiting the highest virulence and multidrug resistance was selected for further characterization**.** Universal primers 27 F and 1492R were used to amplify the full-length 16S rRNA gene sequence^33^. The PCR product was visualized on an agarose gel stained with ethidium bromide (0.5 µg/mL). Sanger sequencing of the PCR amplicon was carried out using an Applied Biosystems 3130 automated DNA Sequencer and a BigDye Terminator V3.1 Cycle Sequencing Kit. The obtained sequence was compared to sequences in the GenBank database using BLAST software to determine the identity of the isolate.

## Experimental animals and experimental design

### Animals and acclimation

#### Clams

Sixty *P. undulata* clams (weighing 10 ± 0.3 g) were collected from Lake Temsah, Ismailia, Egypt, in October 2023. The clams were transported live within two hours in a well-aerated box (80 × 80 × 40 cm) containing 60 L of seawater to the wet laboratory at the Animal Health Research Institute, Zagazig Branch. Upon arrival, the clams were distributed equally into six well-aerated plastic tanks (40 × 40 × 12 cm) containing 3 L of sea mud each. The clams were acclimated for one week at 24 °C with daily feeding of 0.2 g/L spirulina (approximately 1 teaspoonful per tank) after a complete water change.

#### Fish

One hundred twenty healthy red tilapia (*Oreochromis mossambicus* × O. niloticus) with an average body weight of 29 ± 1.89 g were obtained from the Fish Farming and Technology Institute, Suez Canal University, Ismailia, Egypt. The fish were transported live to the wet laboratory at the Animal Health Research Institute, Zagazig branch. They were stocked in 12 glass aquariums (100 × 40 × 40 cm), with 10 fish per aquarium. The fish were fed a commercial diet (30% protein) twice daily at 4% of their body weight throughout the acclimation period.

#### Water quality

Water parameters for both clams and fish were maintained at optimal levels: salinity (20 ppt), dissolved oxygen (6.4 ppm), pH 7.8, ammonia (0.015 ppm), and temperature (24 °C).

### Experimental setup

**Fig. 1:**
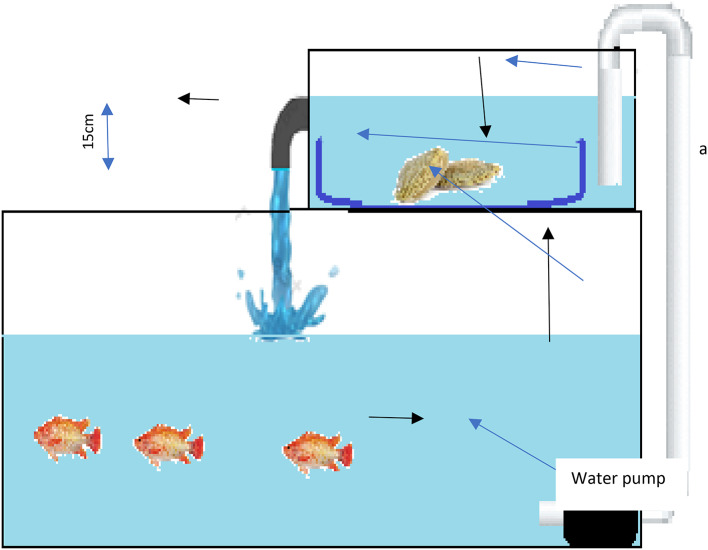
Experimental design: Clam-fish co-culture system.

To initiate the experiment, spirulina feeding was halted 48 h prior to infection. The water and sea mud in the tanks were completely replaced. Red tilapia were divided into four groups, each with three replicates, as detailed in Fig. 1 and Figure S1.

- **Group 1 (Control Negative):** Injected with a 0.9% normal saline and housed in aquariums with plastic tanks containing only sea mud (no clams). This saline concentration is a standard physiological saline solution widely used in aquaculture studies as a negative control, mimicking the vehicle of injection without introducing extraneous substances^34,35^.
- **Group 2 (Infected Control):** Fish were injected intraperitoneally with 1 × 10⁸ CFU/ml of *V. alginolyticus*, and housed in aquariums with plastic tanks containing only sea mud (no clams).
- **Group 3 (Clam-treated Control):** Injected with normal saline and housed in aquariums with shallow glass aquariums containing plastic tanks with clams.
- **Group 4 (Clam-treated Infected):** Fish were injected intraperitoneally with 1 × 10⁸ CFU/ml of *V. alginolyticus* and housed in aquariums with shallow glass aquariums containing plastic tanks with clams.

For the clam-treated groups (Groups 3 and 4), clam tanks were placed within shallow glass aquariums (50 × 40 × 20 cm). Excess water from the shallow aquariums overflowed into the glass aquariums containing the fish, creating a water current without disturbing the sea mud in the plastic tanks. This experimental setup aimed to assess the potential beneficial effects of the clams on water quality and fish health, particularly in the presence of *V. alginolyticus* infection.The controlled water exchange between clam and fish aquariums facilitated the potential transfer of clam-released bioactive compounds, aiming to improve water quality and enhance fish health. The experiment lasted for 16 days, with 7 days for acclimation. The infection was administered on the 9 th day from the beginning. At the end of the experiment, whole blood was collected for hematological analysis, serum was collected for biochemical analysis, and internal organs (liver, kidney, gills, and spleen) were collected for bacteriological, histopathological, and gene expression analyses.

*Observation Period:* The mortalities were recorded for one week after the injection. Therefore, the fish were observed for 7 days after the injection of *V. alginolyticus*.

### Growth performance and clinical observations

To assess the impact of the treatments on fish growth, initial body weight (BW) of each fish was measured at the beginning of the experiment. Body weight and feed intake (FI) were then monitored throughout the study period. At the end of the experiment, final body weight was measured, and growth performance parameters were calculated:

1. Body weight gain (BWG): Calculated as final BW—initial BW.
2. Feed conversion ratio (FCR): Calculated as FI/BWG.

Additionally, clinical signs and mortality rates were recorded daily throughout the experimental period. Deceased fish were necropsied, and macroscopic lesions such as hemorrhages, ascites, and organ abnormalities were recorded and scored based on severity^36,37^.

### Bacteriological examination

To quantify *V. alginolyticus* bacterial loads, serial dilutions of homogenates from the liver, kidney, gills, and spleen of each fish were prepared in sterile saline solution. 0.1 mL aliquots of each dilution were plated onto TCBS agar plates and incubated at 37 °C for 48 h. Following incubation, characteristic yellow, round colonies with a diameter of 2–3 mm, indicative of *Vibrio* species, were enumerated on plates containing 25 to 250 colony-forming units (CFUs) to determine the bacterial load^38^.

### Hematological analysis

Complete blood counts, including hemoglobin concentration, packed cell volume, red blood cell count, white blood cell count, and differential white blood cell count, were determined according to the methods described by^39^.

### Serum biochemical analysis

Serum total protein, albumin, and globulin levels were determined using standard colorimetric methods^40^. Liver enzyme activities, including alanine aminotransferase (ALT) and aspartate aminotransferase (AST), were measured using commercially available kits according to the manufacturer’s instructions^41^. Lactate dehydrogenase (LDH) activity was assessed using a commercially available kit based on the method described by Sameen and Qais Al-Ani^42^. Superoxide dismutase (SOD) activity was measured using a commercially available kit based on the method of Spitz and Oberley^42^. Serum urea and creatinine levels were determined using commercially available kits according to the manufacturer’s instructions^39^. Malondialdehyde (MDA) levels were measured using a commercially available kit based on the method described previously^39^. Serum nitric oxide (NO) and interleukin-6 (IL-6) levels were quantified using commercial ELISA kits (Cat. Nos. MBS723386 and R6000B, respectively). Lysozyme activity was measured using a commercial ELISA kit (Cat. No. MBS725718). Serum immunoglobulin M (IgM) levels were determined using a commercially available kit based on the method described previously^43^.

### Gene expression analysis for quantification of host response and bacterial virulence

Gene expression levels of host response and bacterial virulence were quantified using real-time PCR. Total RNA was extracted from tissue samples using the QIAamp RNeasy Mini Kit (Qiagen, Germany). The average concentration of the extracted RNA was 250 ng/μL, with an average 260/280 ratio of 2.05 and an average 260/230 ratio of 2.1. These values were determined using a NanoDrop Eight Spectrophotometer (ThermoScientific). Real-time PCR was performed using the HERA SYBR® Green RT-qPCR Master Mix (Willowfort, UK) on a StepOne™ real-time PCR system. For host response analysis, β-actin was used as the endogenous control gene for normalization. β-actin is a commonly used and well-validated housekeeping gene in tilapia, exhibiting stable expression across various tissues and under different experimental conditions^44,45^. For the analysis of *V. alginolyticus* virulence genes (*tdh*, *toxR*, and *trh*), 16S rRNA was used as an internal control, leveraging its stable and universal expression in bacteria. The virulence genes targeted encode critical pathogenicity factors: *tdh* for thermostable direct hemolysin, *toxR* for a transcriptional regulator of virulence genes, and *trh* for TDH-related hemolysin. Primer sequences and PCR conditions are listed in Tables 1–2. A 20 µL reaction mixture was prepared containing 10 µL of 2 × HERA SYBR® Green RT-qPCR Master Mix, 1 µL of RT Enzyme Mix (20X), 0.5 µL of each primer (20 pmol), 3 µL of water, and 5 µL of RNA template. A no-template control was included in each PCR run to ensure the absence of contamination. Relative gene expression levels were calculated using the 2-^ΔΔCt^ method, which normalizes target gene expression to the respective internal control gene and determines fold changes between experimental and control groups^46,47^.

**Table 2:** Primers sequences and cycling conditions used for RT-qPCR analysis of host response gene expression.

Target gene	Primers sequences	Annealing (°C)	Reference
β*- actin*	F; CGAG CAGGAGATGG GAACC	60	^48^
	R; CAACGGAAACG CTCATTGC		
*PACK*	F; GAGAATTCTCACACACAC ACACGTGAGCAGTA	55	^49^
	R; GTAAAAGCTTTCCGCCATAACATCTCCAGC A GAA		
*Tfam*	F; GGCAAGTTGTCCAAAGAAACC	55	^50^
	R; GCATCTGGGTTCTGAGCTTTA		
*SOD*	F; TCCGCACTTCAACCCTCA	60	^51^
	R; CCTCATTGCCACCCTTCC		
*CAT*	F; TACCAGTCAACTGCCCGTAC	60	^51^
	R; GACTCAAGGAAGCGTGGC		
*GST*	F; CCAACCACCTCAAATGCT	60	^51^
	R; ACGGGAAAGAGTCCAGACAG		
*IL-1β*	F; CCTCTCCTCAAACCTTCAGACC	60	^52^
	R; TGCTGTGTTTGATGTCGTTCAC		
*IL-6*	F; CAGCAGAATGGGGGAGTTATC	60	^53^
	R; CTCGCAGAGTCTTGACATCCTT		
*IL-18*	F; GCTTGAATCTAAATTATCAGTC	60	^54^
	R; GAAGATTCAAATTGCATCTTAT		
*IL-22*	F; CCGTACTGTAGCAACAGTGCAG	60	^55^
	R; TCACATTCTTGCAGAGCAGGATTC		
*IL-10*	F; GCAACAGAACATCAATAGTCCTT	60	^53^
	R; CACCCTTTTCCTTCATCTTTTCA		
*IL-34*	F; TCAACAGGGTATAAAGAGGGTT	60	^53^
	R; ATCCAGTAATGACTTGGGTGTA		
*TGF-β1*	F; TTGGGACTTGTGCTCTAT	60	^53^
	R; AGTTCTGCTGGGATGTTT		
*IFN-γ*	F; CTCTTGGCTGTTACTGCCAGG	60	^56^
	R; CTCCACACTCTTTTGGATGCT		

### Histopathological examination

Fresh tissue samples from the gills, liver, spleen, and intestine of fish from each experimental group were collected and fixed in 10% neutral-buffered formalin. The tissues were then processed through dehydration, clearing, and paraffin embedding. Paraffin Sects. (5–7 µm thick) were cut using a Slee CUT 4062 rotary microtome (Slee Medical GmbH, Germany). The sections were stained with hematoxylin and eosin and examined under a light microscope^57^.

### Preparation of *P. undulata* methanolic extract and in vitro antimicrobial activity assessment

Bioactive compounds were extracted from dried and powdered *P. undulata* using methanol^58^. Methanol was selected as the extraction solvent due to its high polarity and ability to effectively extract a wide range of polar compounds, including phenols, flavonoids, and terpenoids, commonly found in marine organisms and known for their diverse biological activities^59–61^. *P. undulata* samples were soaked in methanol for 24 h at room temperature, followed by sonication for 1 h at 40 °C to enhance extraction. The extract was filtered through Whatman No. 1 filter paper and concentrated using a rotary evaporator.

To assess the antimicrobial activity of the *P. undulata* extract, a highly virulent, drug-resistant *V. alginolyticus* isolate was selected. The agar well diffusion method was employed to determine the antibacterial activity. Bacterial cultures were standardized to a 0.5 McFarland turbidity standard and swabbed onto Mueller–Hinton agar plates. Wells were created in the agar, and 10 µL of the *P. undulata* extract (100% v/v) was added to each well. Sterile water served as a negative control. The plates were incubated at 37 °C for 24 h, and the diameters of the inhibition zones were measured.

The minimum inhibitory concentration (MIC) of the *P. undulata* extract was determined using a broth microdilution assay according to CLSI^27^ guidelines. Serial dilutions of the extract (32 to 0.0625 µg/mL) were prepared in Mueller–Hinton broth, and a standardized bacterial suspension was added to each well. The lowest concentration of the extract that inhibited bacterial growth was considered the MIC.

### GC–MS analysis of *P. undulata* extract

Chemical composition analysis of extract was performed using a Trace GC1310-ISQ mass spectrometer (Thermo Scientific, Austin, TX, USA) equipped with a TG5MS capillary column (30 m × 0.25 mm i.d., 0.25 µm film thickness). The oven temperature was programmed as follows: initial temperature of 35 °C held for 5 min, ramped to 200 °C at a rate of 3 °C/min, held for 3 min, then ramped to 280 °C at a rate of 3 °C/min and held for 10 min. The injector and MS transfer line temperatures were maintained at 250 °C and 260 °C, respectively. Helium was used as the carrier gas at a constant flow rate of 1 mL/min. One microliter of diluted sample was injected automatically using an AS1300 autosampler in split mode. Electron ionization (EI) mass spectra were acquired at 70 eV over an m/z range of 40–1000 in full scan mode. The ion source temperature was set at 200 °C. Compound identification was performed by comparing their retention times and mass spectra with those in the NIST 11 and Wiley 09 mass spectral libraries.

### Fourier transform infrared (FTIR) spectroscopy

FTIR spectroscopy was employed to further characterize the chemical composition of the *P. undulata* extract. Approximately 150 mg of the extract was prepared as a KBr pellet and analyzed using an FTIR spectrometer. The resulting spectrum was analyzed to identify functional groups present in the extract. The characteristic absorption bands observed in the FTIR spectrum provided insights into the molecular structure of the compounds present in the extract. By comparing the obtained spectrum with standard reference spectra, it was possible to confirm the presence of various functional groups, such as hydroxyl, carbonyl, and aromatic groups, which are associated with the identified compounds in the GC–MS analysis.

### Statistical analysis

Data were analyzed using Microsoft Excel and SPSS software (version 2004). Hematological parameters were analyzed using ANOVA, followed by Duncan’s Multiple Range Test. Prior to ANOVA, normality was assessed using the Shapiro–Wilk test, and homogeneity of variance was assessed using Levene’s test. Normality of biochemical parameters and gene expression data was assessed using the Shapiro–Wilk test. One-way ANOVA was employed to determine significant differences among treatment groups, followed by Tukey’s HSD test for pairwise comparisons. Chi-square tests were used to analyze resistance patterns, and logistic regression was used to assess the impact of sample source on *Vibrio* prevalence. All statistical analyses were performed at a significance level of α = 0.05. Effect sizes, such as Cohen’s d, will be calculated and reported for the ANOVA results on hematological parameters. Figures were generated using GraphPad Prism software 9.0 (GraphPad, United States).

## Results

### Occurrence and molecular identification of *Vibrio alginolyticus*

Morphological and biochemical characterization identified *Vibrio* spp. in 22% of the examined fish samples. Among these, 12% were confirmed as *V. alginolyticus* based on their characteristic yellow colony morphology on TCBS agar, Gram-negative, short bacillary shape, positive oxidase and catalase tests, tolerance to 6% NaCl, and positive indole production, negative citrate utilization, negative urease, negative triple sugar iron, negative gelatin hydrolysis, and positive methyl red tests**.** Molecular identification using species-specific gene (collagenase gene) PCR confirmed the identity of these isolates, with a characteristic band size of 738 bp(Figure S2).

### Antimicrobial susceptibility testing, genomic characterization, and sequencing Data

Antimicrobial susceptibility testing revealed a high prevalence of multidrug resistance among 12 V*. alginolyticus* isolates. Notably, 11 isolates exhibited resistance to ampicillin, doxycycline, and erythromycin, while 4 demonstrated an extreme drug-resistant (XDR) phenotype. Conversely, high sensitivity was observed to oxolinic acid and trimethoprim/sulfamethoxazole (8 out of 12), and moderate sensitivity to oxytetracycline (5 out of 12)(Figure S3). The Multiple Antimicrobial Resistance Index (MARI) ranged from 0.14 to 1.0 (Table S1).

Genomic characterization revealed the presence of virulence genes, with *tdh* being the most prevalent (58.33%), followed by *toxR* (50%) and *trh* (8.3%). Statistical analysis indicated a significant association between resistance to antimicrobial agents and the presence of the *trh* gene (p < 0.05, Figure S4).

A highly virulent, pan-resistant *V. alginolyticus* isolate, characterized by its resistance to multiple antimicrobial agents and the presence of virulence genes, was selected for further analysis and challenge. The 16S rRNA gene sequence of this isolate was deposited in GenBank under accession number PQ516971.

### Experimental model findings following *V. alginolyticus* challenge

This section presents the results obtained from red tilapia following challenge with *V. alginolyticus*, unless otherwise stated for control groups. The effects of dietary supplementation with *P. undulata* on growth performance, mortality, bacterial load, hematological parameters, biochemical markers, gene expression, clinical observations, and histopathological findings were evaluated.

1. Growth performance and mortality of red tilapia following *V. alginolyticus* challenge

Dietary supplementation with *P. undulata* significantly enhanced growth performance in red tilapia challenged with *V. alginolyticus*, as evidenced by increased final weight, weight gain, and specific growth rate (SGR) and improved feed conversion ratio (FCR) compared to the infected control group (G2) (Table 3). Notably, these positive growth parameters were maintained even in the presence of *V. alginolyticus* infection.

**Table 3:** Effect of *P. undulata* on growth performance of red tilapia.

Items	Groups				***p***-values
	G1	G2	G3	G4	
Initial weight (g)	38.04 ± 2.27	38.42 ± 1.56	38.22 ± 1.44	38.22 ± 1.61	0.9947
Final weight (g)	45.00 ± 1.50	40.60 ± 1.20	48.30 ± 1.56	46.60 ± 1.89	0.012
Weight gain (g)	7.96 ± 1.02	2.18 ± 0.98	10.08 ± 1.12	8.38 ± 1.32	0.035
Feed Conversion Ratio (FCR)	1.85 ± 0.12	2.12 ± 0.25	1.58 ± 0.18	1.72 ± 0.21	0.042
Specific Growth Rate (SGR)	85.23 ± 2.11	72.45 ± 1.98	90.12 ± 1.35	87.65 ± 2.34	0.028

**Fig. 2:**
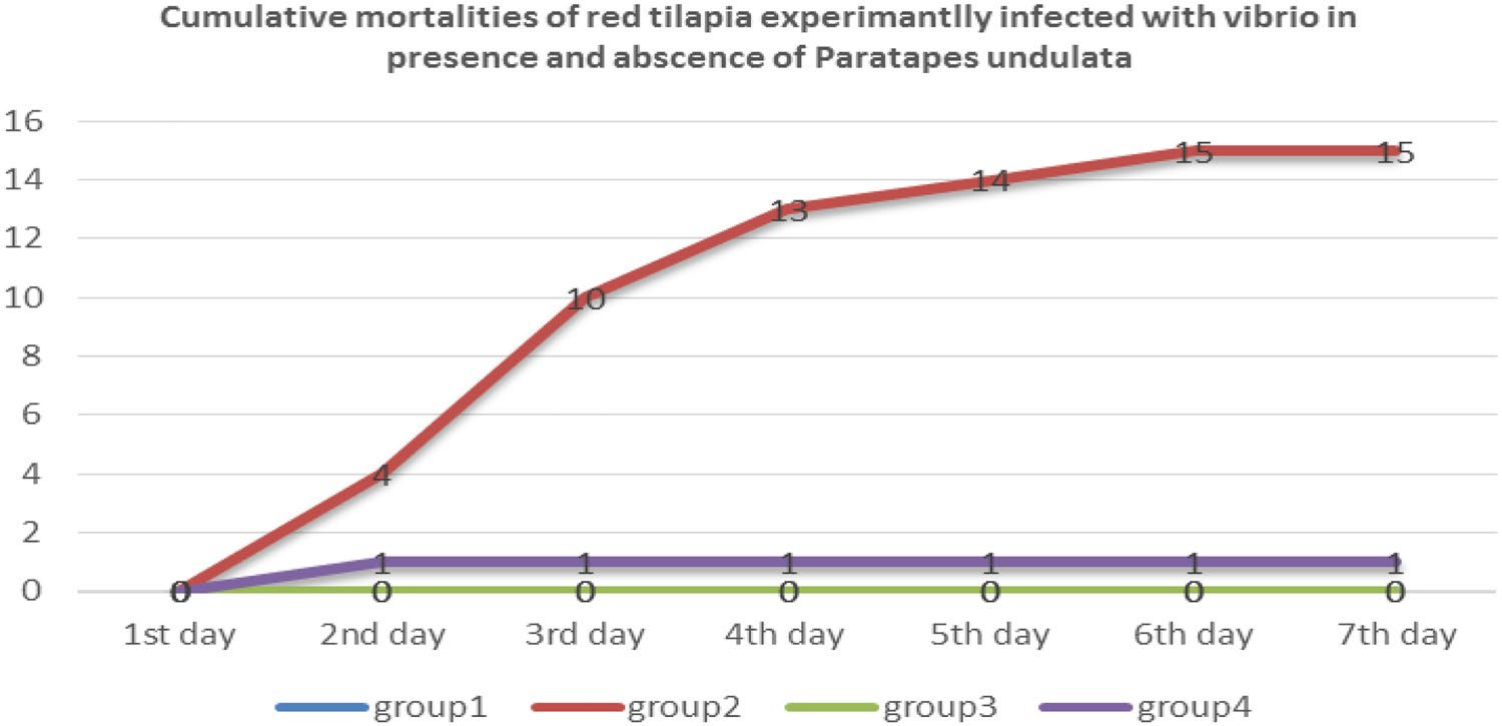
Cumulative mortality of red tilapia (%).

Following challenge with *V. alginolyticus*, cumulative mortality rates were significantly lower in *P. undulata*-treated groups (G3 and G4) compared to the infected control group (G2). Mortality peaked on the third day post-infection in the infected control group and gradually declined thereafter. In contrast, no mortalities were observed in the saline-injected control group (G1) and the clam-treated control group (G3) (Fig. 2).

2. Bacteriological evaluation following *V. alginolyticus* challenge

**Fig. 3:**
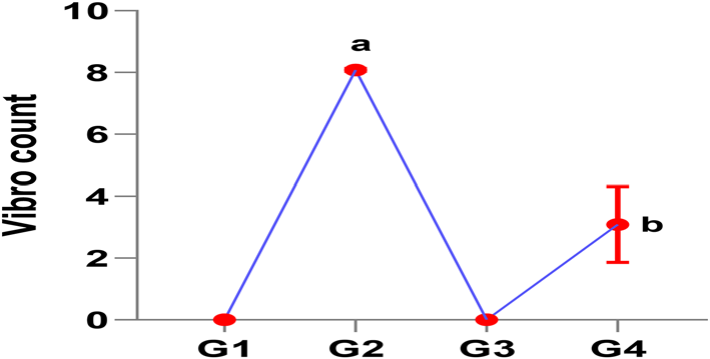
Enumeration of *V. alginolyticus* in different groups.

Figure 3 illustrates the significant reduction in *Vibrio* counts determined from homogenates of the internal organs in the clam-treated groups (G3 and G4) compared to the infected control group (G2). Notably, *Vibrio* counts were completely eliminated in both the saline-injected control group (G1) and the clam-treated control group (G3).

3. Hematological findings following *V. alginolyticus* challenge

*V. alginolyticus* infection significantly impacted hematological parameters in the infected control group (G2), resulting in a significant decrease in red blood cell (RBC) count, hemoglobin concentration, and packed cell volume compared to the saline control group (G1). This was accompanied by leukocytosis, lymphocytosis, neutrophilia, and monocytosis, indicative of an inflammatory response. In contrast, the infected-treated group (G4) exhibited a significant improvement in these parameters, with increased RBC count, hemoglobin concentration, and packed cell volume. Concurrently, leukocyte counts, including lymphocytes, neutrophils, and monocytes, were significantly reduced in G4 compared to the infected control group (Table 4).

**Table 4:** Hemogram of vibrio infected and treated fish (M ± SE).

Item	RBCs (10^6^/mm^3^)	Hb (g/dl)	PCV %	WBCS (10^3^ × mm^3^)	Lymphocytes (10^3^ × mm^3^)	Neutrophil (10^3^ × mm^3^)	Monocytes (10^3^ × mm^3^)
G1	5.22 ± 0.04^a^	10.38 ± 0.08^ab^	31.33 ± 0.21^a^	11.26 ± 0.29^c^	6.11 ± 0.12^c^	4.65 ± 0.15^bc^	0.45 ± 0.02^b^
G2	2.66 ± 0.21^b^	8.21 ± 0.14^c^	24.67 ± 0.3^b^	15.04 ± 0.18^a^	9.05 ± 0.28^a^	5.25 ± 0.34^a^	0.66 ± 0.05^a^
G3	5.24 ± 0.04^a^	10.55 ± 0.17^a^	31.64 ± 0.23^a^	11.4 ± 0.43^c^	6.51 ± 0.04^bc^	4.42 ± 0.26^c^	0.43 ± 0.02^b^
G4	4.9 ± 0.09^a^	9.59 ± 0.23^b^	30.1 ± 0.31^a^	12.49 ± 0.46^b^	6.76 ± 0.15^b^	5.1 ± 0.13^ab^	0.57 ± 0.03^a^

RBCs: red blood cells; Hb: hemoglobin; PCV: packed cell volume; WBCS: white blood cells. Different letters in the same column indicate significant changes.

4. Biochemical findings

**Fig. 4:**
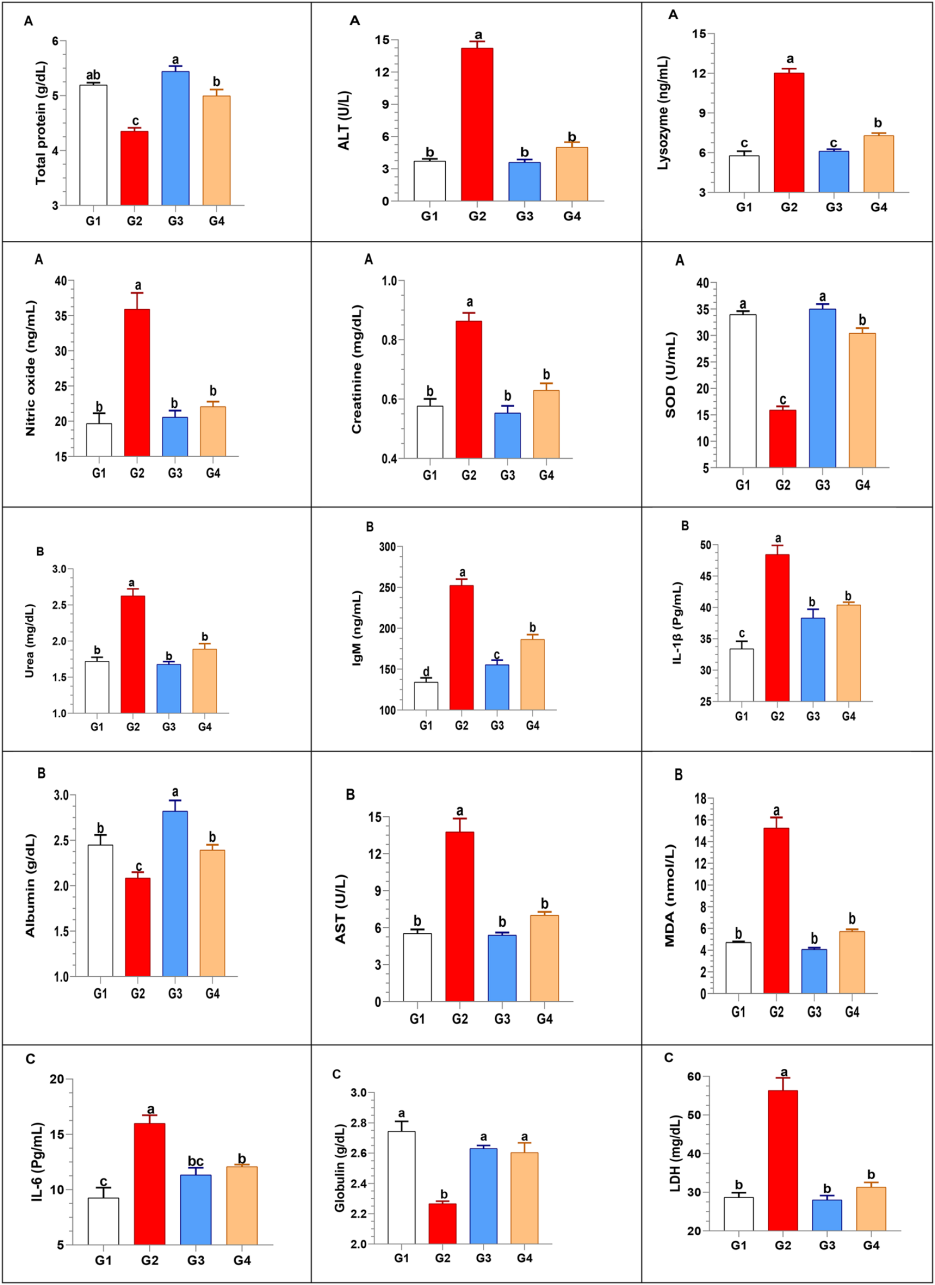
Effect of *P. undulata* on biochemical parameters in red tilapia.

As shown in Fig. 4, the levels of total protein and its fractions were significantly elevated in the clam-treated groups (G3 and G4) compared to the infected control group (G2). Regarding liver function, the activities of liver enzymes, including alanine aminotransferase (ALT), aspartate aminotransferase (AST), and lactate dehydrogenase (LDH), were significantly elevated in the infected control group (G2). This elevation of liver enzymes is a direct indicator of liver damage and cellular leakage, reflecting the severity of the *V. alginolyticus* infection. However, these levels were significantly reduced in both clam-treated groups (G3 and G4), with the most significant reduction observed in G3. This reduction suggests that the *P. undulata* supplementation effectively mitigated liver damage and reduced the severity of the infection.

Kidney function, as assessed by creatinine and urea levels, was significantly impaired in the infected control group (G2). In contrast, both clam-treated groups (G3 and G4) exhibited significantly lower levels of these biomarkers, indicating improved kidney function.

For immune parameters, lysozyme activity, nitric oxide levels, and IgM levels were significantly elevated in the infected control group (G2). However, these parameters were significantly decreased in both clam-treated groups, indicating a reduced inflammatory response and improved immune function.

The antioxidant status, as assessed by superoxide dismutase (SOD) and malondialdehyde (MDA) levels, was well-maintained in both the control and clam-treated groups (G1 and G3). In contrast, the infected control group (G2) exhibited increased oxidative stress, as indicated by higher MDA levels.

Furthermore, the levels of pro-inflammatory cytokines, IL-1β and IL-6, were significantly lower in the control and clam-treated groups (G1 and G3) compared to the infected control group (G2).

5. Gene expression analysis

To investigate the underlying mechanisms of the protective effects of *P. undulata* extract, the expression levels of various genes involved in antivirulence, metabolic, oxidative stress, and immune responses were assessed using qRT-PCR in the Biotechnology Unit, Animal Health Research Institute, Zagazig Branch, Egypt.

### Host gene expression

#### Antioxidant response

**Fig. 5:**
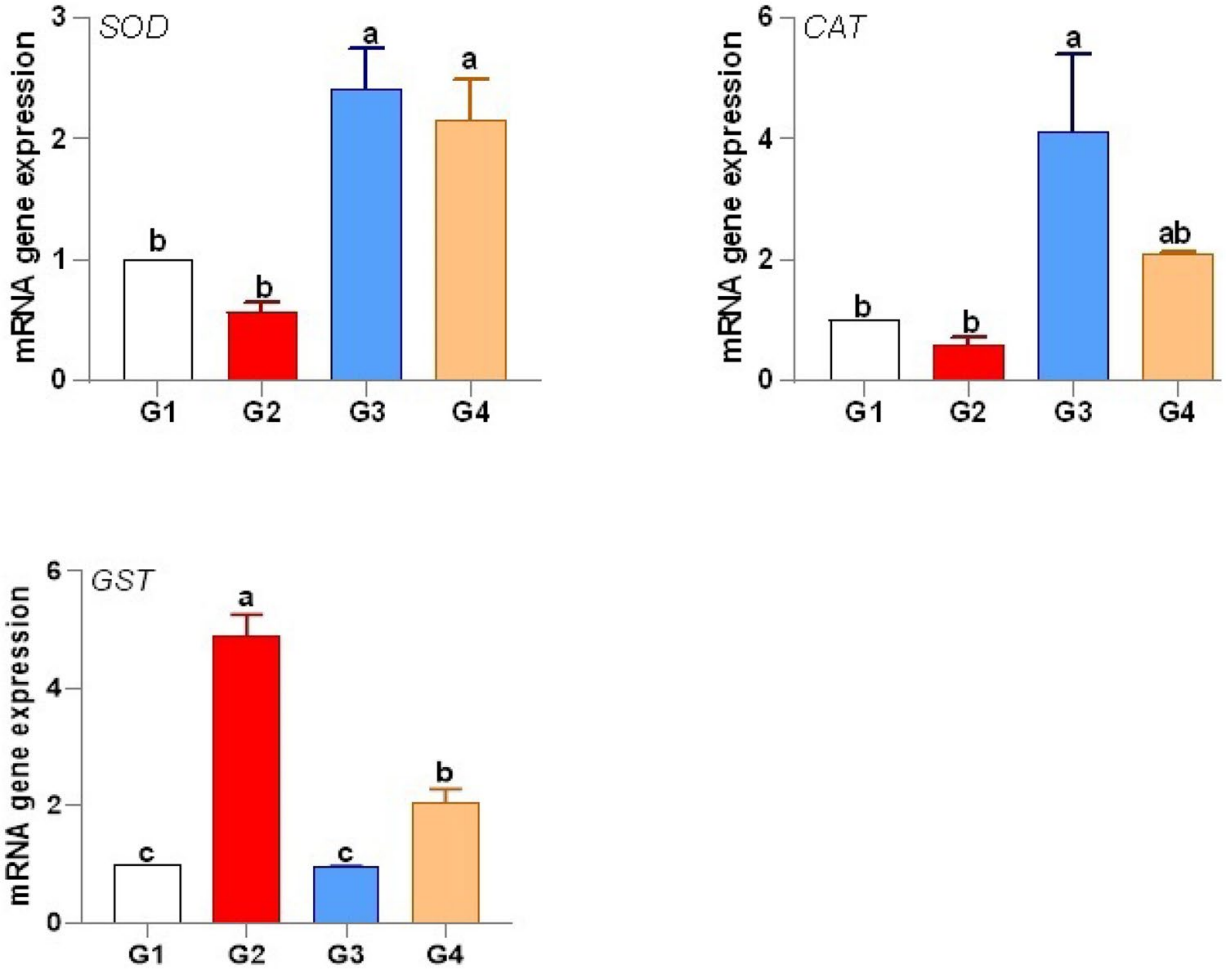
Modulation of antioxidant gene expression in *P. undulata*-treated fish.

*P. undulata* supplementation significantly enhanced the antioxidant capacity of fish challenged with *V. alginolyticus* (Fig. 5). The expression of antioxidant genes, superoxide dismutase (*SOD*) and catalase (*CAT*), was significantly upregulated in the *P. undulata*-supplemented infected group (G4) compared to the infected control group (G2), by 2.16-fold and 2.08-fold, respectively. This upregulation indicates an enhanced ability to neutralize reactive oxygen species (ROS). Conversely, the expression of glutathione S-transferase (*GST*) was significantly downregulated in G4 by 2.04-fold. This downregulation, while seemingly contradictory, may reflect a shift in the antioxidant defense strategy. With *SOD* and *CAT* effectively scavenging ROS, the need for *GST*-mediated detoxification of lipid peroxides may be reduced. Alternatively, this could indicate a specific modulation of *GST* isoforms, which warrants further investigation. Further studies including SOD1 and other isoforms of SOD would be beneficial to fully elucidate the antioxidant response.

#### Metabolic response

**Fig. 6:**
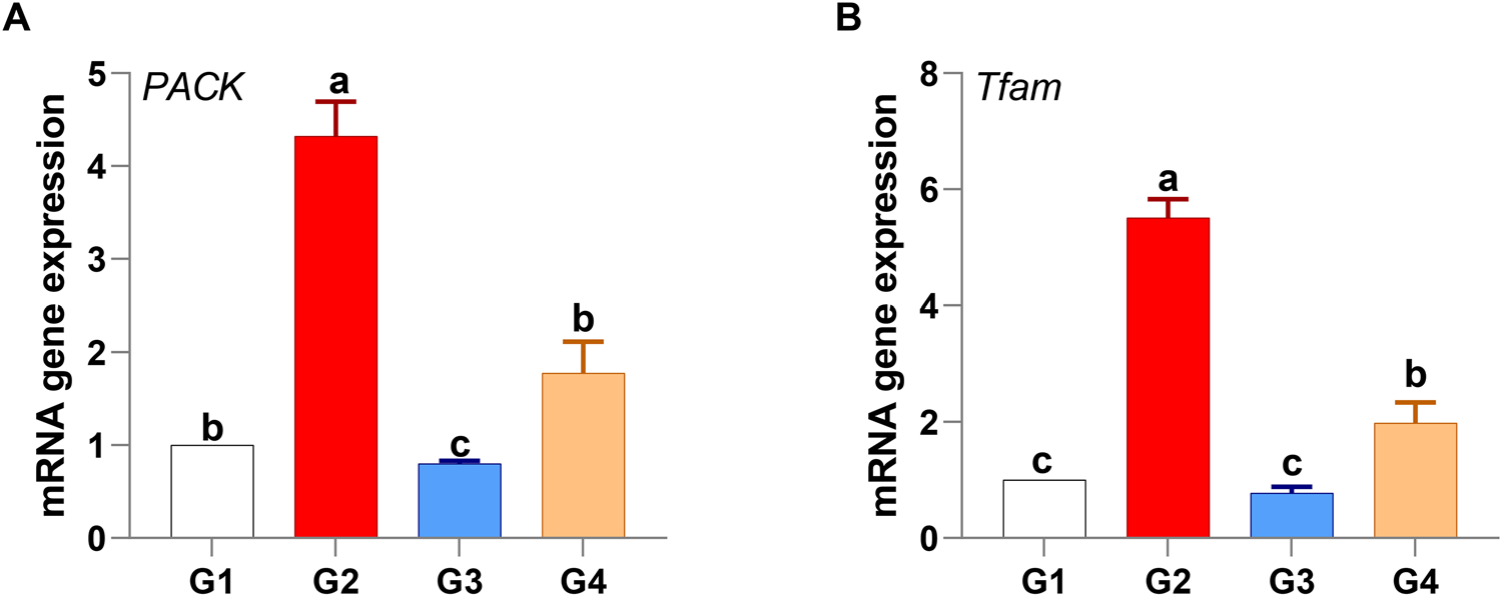
Modulation of metabolic gene expression in *P. undulata*-treated fish.

In response to *V. alginolyticus* infection, *P. undulata* supplementation significantly modulated host metabolism. Specifically, the metabolic genes phosphoenolpyruvate carboxykinase (*PACK*) and mitochondrial transcription factor A (*TFAM*) were significantly downregulated in the *P. undulata*-supplemented group (G4) compared to the infected control group (G2), with fold changes of 1.78 and 1.98, respectively (Fig. 6). This downregulation suggests a strategic metabolic shift, likely prioritizing immune function.

The observed decrease in *PACK* expression indicates a potential reduction in gluconeogenesis. This redirection of metabolic resources may conserve energy that would otherwise be used for glucose synthesis, making it available for immune cell proliferation and activation. Furthermore, limiting glucose production can help mitigate excessive inflammation, a key aspect of effective immune regulation.

Similarly, the downregulation of *TFAM*, a key regulator of mitochondrial DNA transcription, suggests a potential modulation of mitochondrial function. This likely serves to balance energy production with the need to minimize oxidative stress and optimize immune responses. By controlling mitochondrial activity, the host may limit the production of reactive oxygen species (ROS), which can negatively impact immune function and contribute to immunopathology. Additionally, the modulation of mitochondrial dynamics through TFAM downregulation could play a role in regulating inflammasome activation, thus influencing the inflammatory response.

Taken together, the observed downregulation of *PACK* and *TFAM* in *P. undulata*-supplemented tilapia suggests a coordinated metabolic adaptation aimed at optimizing resource allocation for immune defense and minimizing infection-induced stress.

#### Immune response

**Fig. 7:**
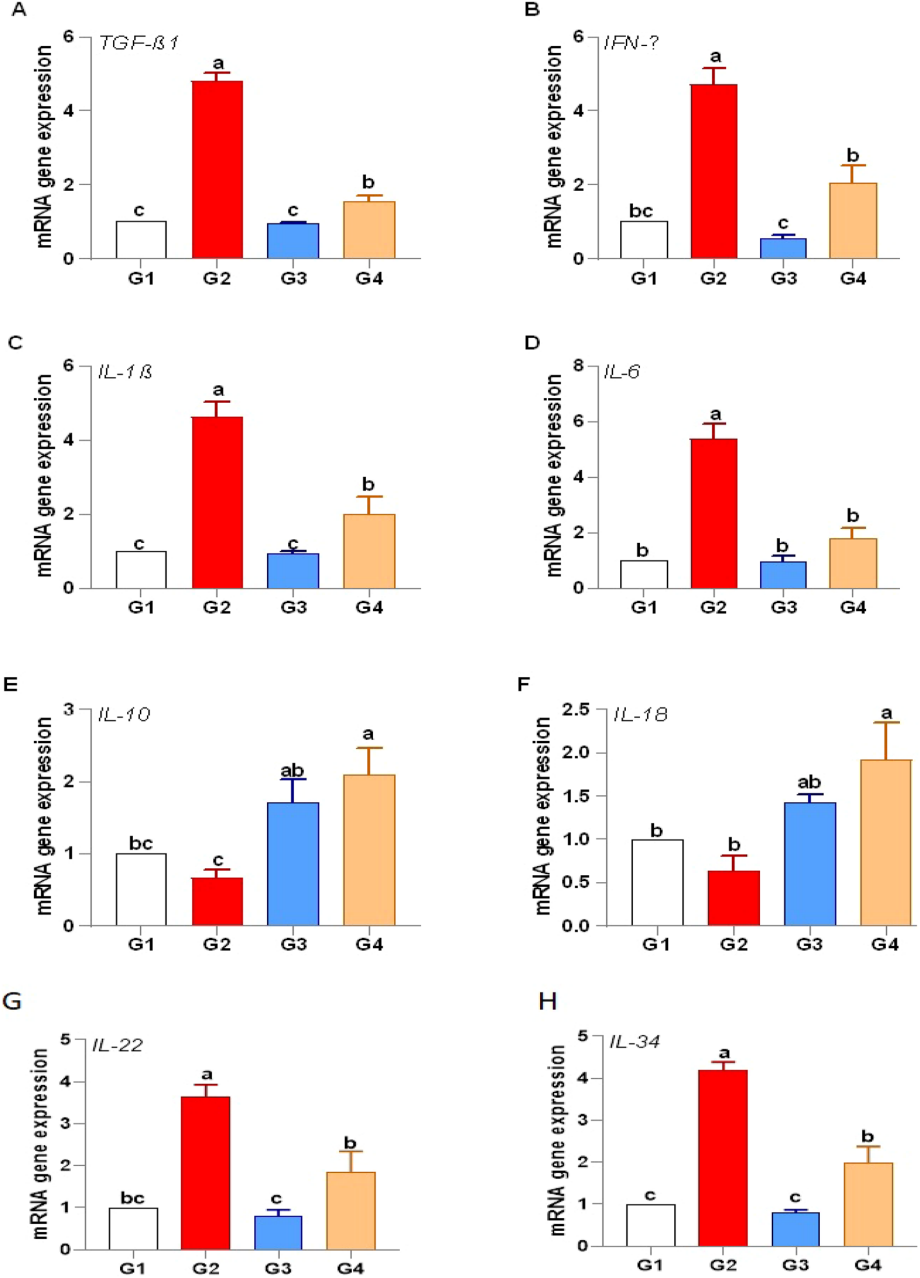
Immune gene expression in response to *P. undulata* treatment.

*P. undulata* supplementation effectively modulated the immune response in fish challenged with *V. alginolyticus*. The expression of pro-inflammatory cytokines, including interleukin-1β (*IL-1β*), *IL-6*, *IL-22*, *IL-34*, and tumor necrosis factor-alpha (*TNF-α*), was significantly upregulated in the infected control group (G2) compared to the control group (G1), indicating an inflammatory response to the pathogen. However, the expression of these cytokines was significantly downregulated in the *P. undulata*-supplemented groups (G3 and G4), suggesting a suppression of excessive inflammation. Conversely, the expression of anti-inflammatory cytokines, *IL-10* and *IL-18*, was significantly upregulated in the *P. undulata*-supplemented groups (G3 and G4) compared to the infected control group (G2), by 2.10-fold and 1.91-fold, respectively, indicating a shift towards immune homeostasis. The expression of immune regulatory cytokines, transforming growth factor-beta 1 (*TGF-β1*) and interferon-gamma (*IFN-γ*), was significantly downregulated in the *P. undulata*-supplemented groups (G3 and G4) compared to the infected control group (G2) by 1.54-fold **(**Fig. 7**)**. This downregulation of *TGF-β1* and *IFN-γ*, in conjunction with the modulation of pro- and anti-inflammatory cytokines, suggests a refined immune response aimed at controlling the infection without triggering excessive inflammation. The upregulation of *SOD* and *CAT* may also play a role in modulating the inflammatory response, as ROS can act as signaling molecules in inflammation. The enhanced antioxidant capacity could therefore contribute to the observed downregulation of pro-inflammatory cytokines.

### Bacterial virulence gene expression

**Fig. 8:**
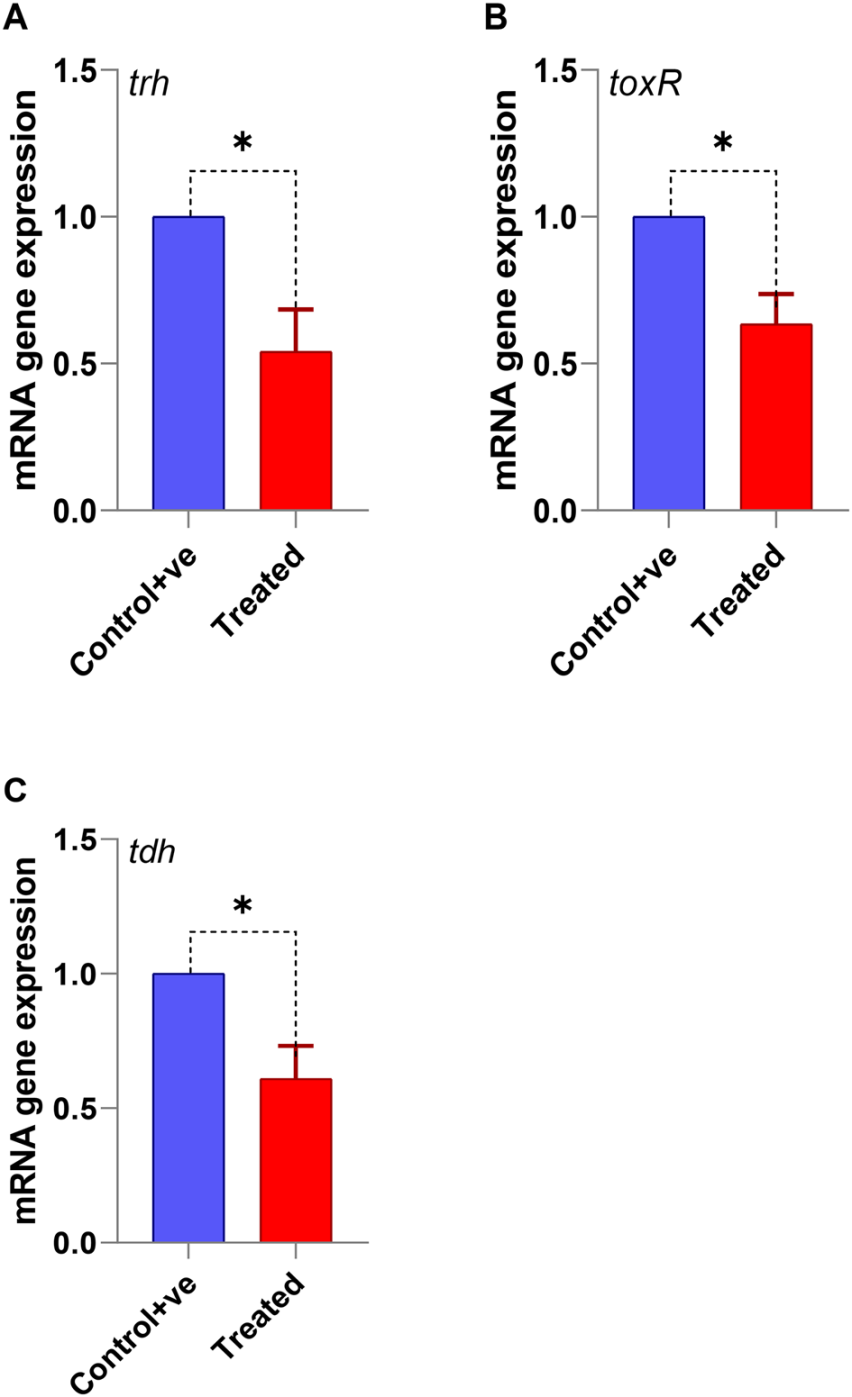
The transcription of all virulence genes including *trh, toxR*, and *tdh* was significantly lower in treated group compared to the positive control (p < 0.05).

Treatment of *V. alginolyticus* with *P. undulata* significantly downregulated the expression of virulence genes, including *tdh*, *trh*, and *toxR*. This finding indicates that bioactive compounds within *P. undulata* may interfere with the expression of bacterial virulence factors, thereby potentially reducing bacterial pathogenicity (Fig. 8).

6. Clinical observations and gross lesions

Fish in the control groups (G1: saline-injected, G3: clam-exposed control) remained clinically healthy throughout the observation period with no observable gross lesions. In contrast, fish in the infected control group (G2) exhibited a range of clinical signs including lethargy, anorexia, erratic swimming behavior, and mild skin irritation. Grossly, these fish displayed diffuse fin erosion, gill congestion, and hepatic and splenic vascular congestion. Some fish also exhibited mild hemorrhagic foci in the liver and spleen.

7. Histopathological findings

**Fig. 9:**
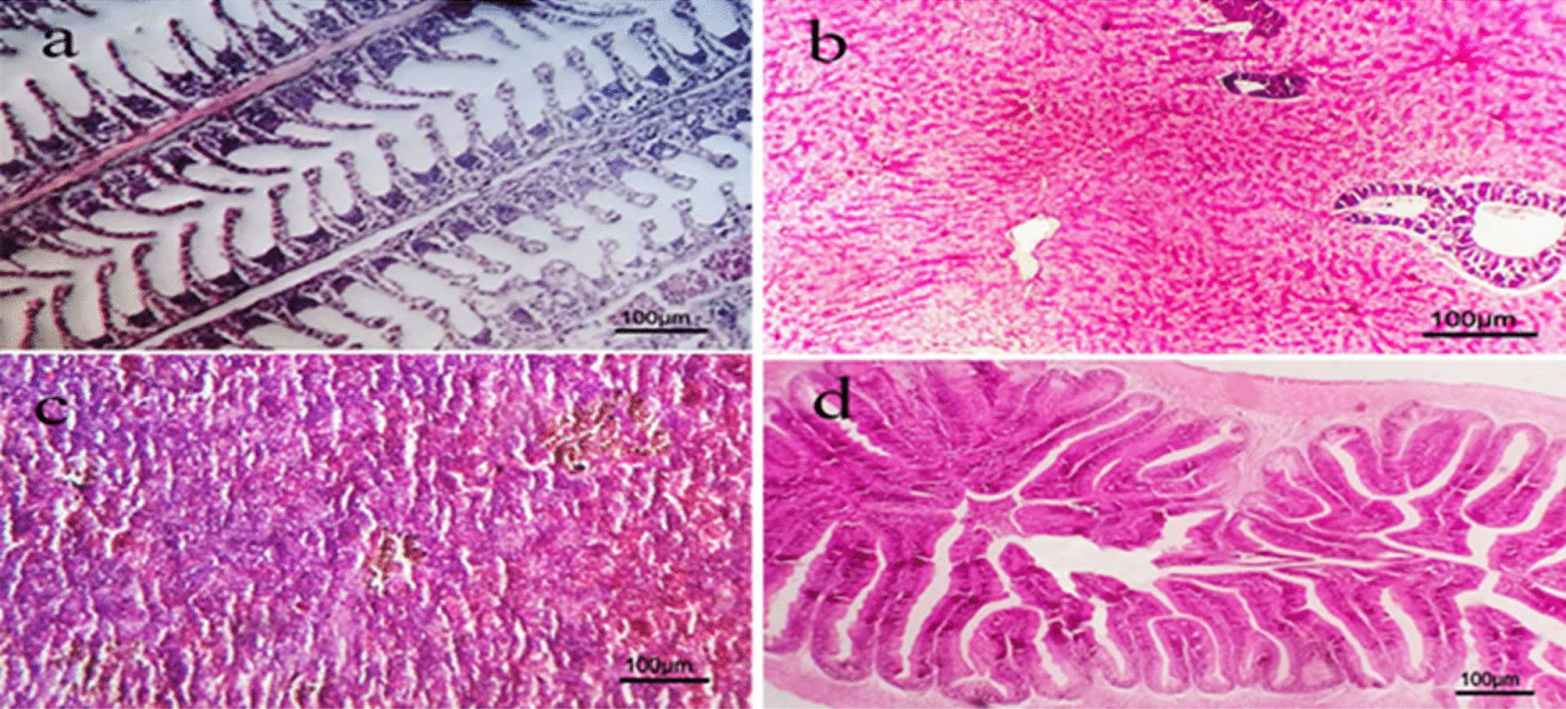
Photomicrograph of H&E section of different organs of control negative group showing: **a)** gills with normal primary and secondary lamellae. **b)** liver with normal hepatic parenchyma and cellular details.**c)** spleen with normal parenchyma white and red pulp. **d)** intestine with normal mucosa, submucosa and serosa. (Scale bar = 100 µm).

**Fig. 10:**
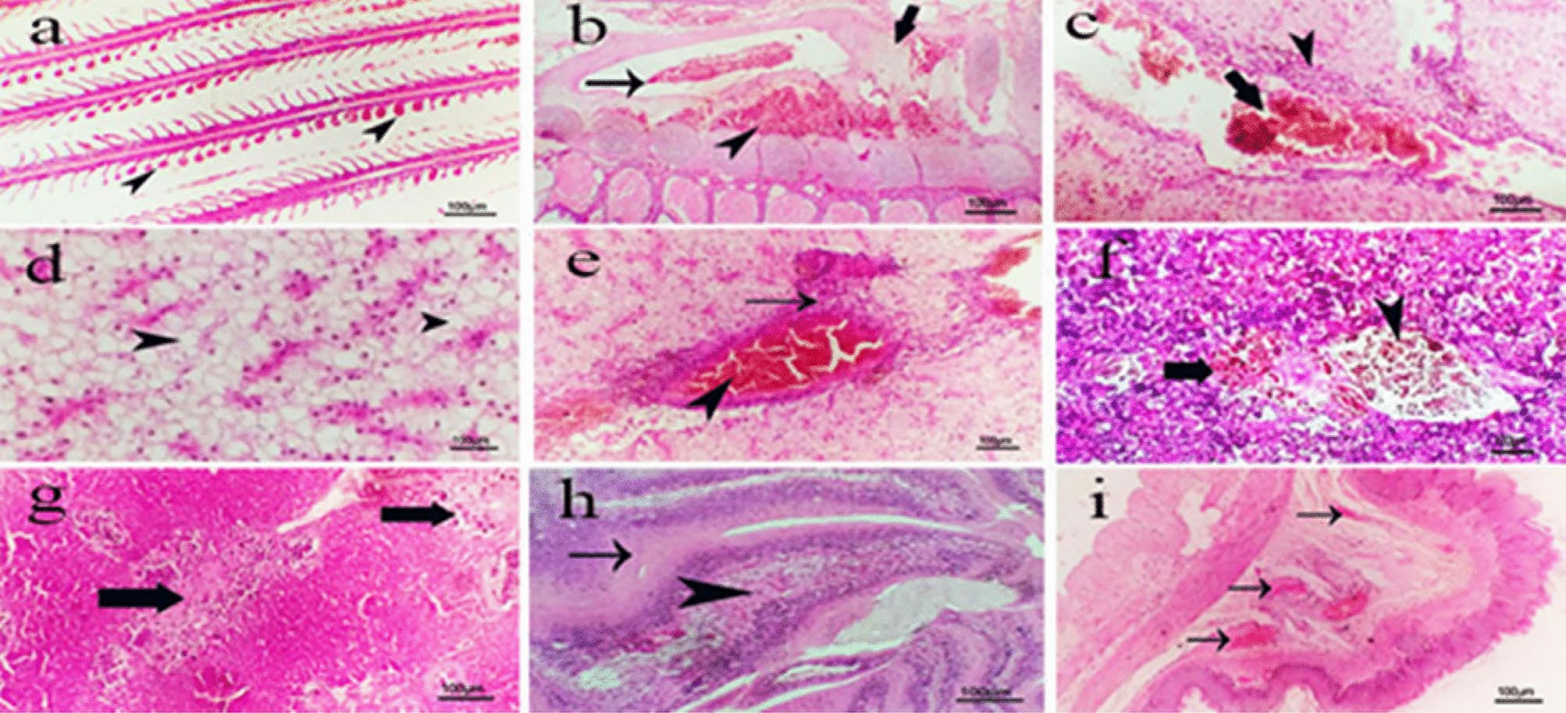
Photomicrograph of H&E section of different organs of red tilapia infected with *V. alginolyticus* showing: **a)** gills with diffuse telangiectasis of secondary lamellae (arrows head) with deformities of others. **b)** gills with severe congestion of blood vessels (thin arrow) with partial fibrosis (thick arrow) and hemorrhage in the gill arch (arrowhead). **c)** liver with severe vascular congestion with extravasated erythrocytes (thick arrow) with perivascular mononuclear inflammatory cells infiltration (arrowhead). **d)** liver with diffuse vacuolation of hepatocytes of mainly fatty degeneration (arrows head). **e)** liver with perivascular inflammatory mononuclear cells infiltration (arrow) and severe vascular congestion (arrow head). **f)** spleen with severe vascular congestion (arrow head) and focal extravasated erythrocytes (hemorrhage) (thick arrow). **g)** spleen with focal fibrosis surrounded with inflammatory reaction (thick arrows). **h)** intestine with moderate focal extravasated erythrocytes in the lamina propria (arrow head) and focal fibrosis of submucosa (arrow). **i)** intestine with diffuse congestion of submucosal blood vessels (arrows). (Scale bar = 100 µm).

**Fig. 11:**
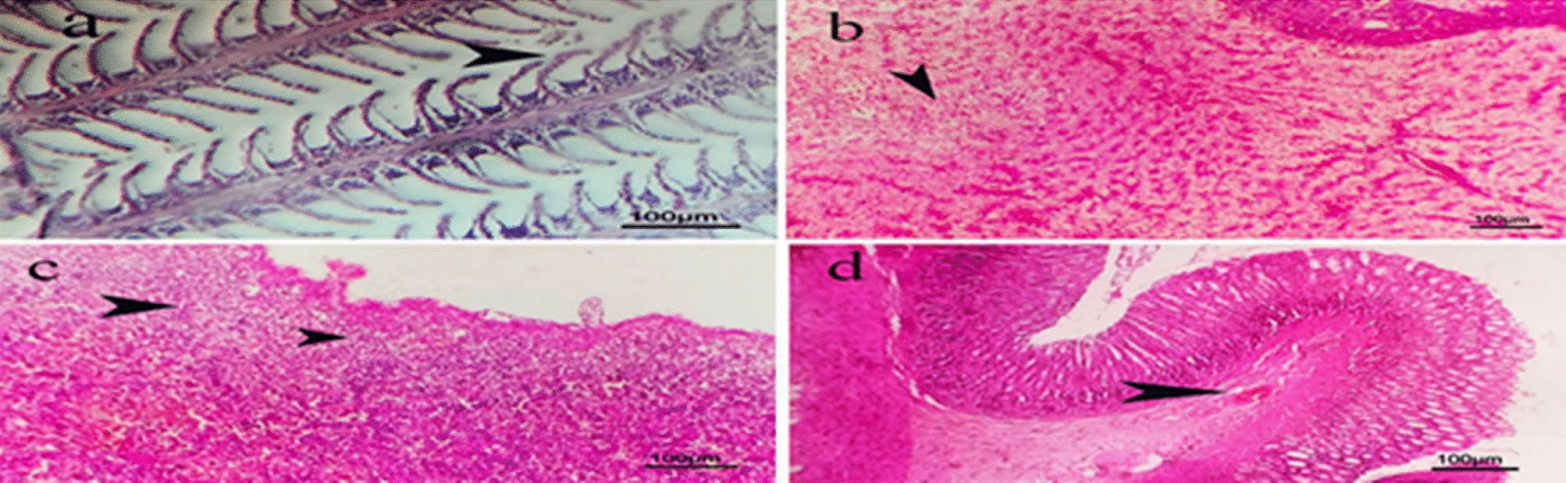
Photomicrograph of H&E section of different organs of red tilapia subjected to *P. undulatus* showing: **a)** gills with apparently normal elongated primary lamellae with normal gill arch and secondary lamellae. **b)** liver with apparently normal hepatic parenchyma and cellular details except in focal mild cellular vacuolation of mainly hydropic degeneration (arrow head). **c)** spleen with normal parenchyma and mild subcapsular lymphocytic cellular depletion (arrows head). **d)** intestine with normal mucosa, submucosa and serosa except in mild submucosal vascular congestion (arrow head). (Scale bar = 100 µm).

Some examined organs of control non infected non treated group (group 1) showed normal gills’ structure, normal gill arch, primary and secondary lamellae **(**Fig. 9a**)**. Liver exhibited normal hepatic parenchymatous architecture and cellular details **(**Fig. 9b**)** while spleen showed normal parenchyma, white and red pulp **(**Fig. 9c**)**. Intestine appeared with normal mucosa, submucosa and serosa** (**Fig. 9d**)**. Red tilapia infected with *V. alginolyticus* **(group 2)** demonstrated variable severe histological changes in different organs such gills exhibited diffuse telangiectasis of secondary lamellae represented in severe vascular dilation with over distended tips with blood in addition to deformities of few other gills include bending fusion and torticollis **(**Fig. 10a**),** severe congestion of blood vessels with partial fibrosis represented in severe to moderate focal proliferation of fibroblast cells producing massive fibers and hemorrhage or extravasated blood oozing out to the gill arch were demonstrated **(**Fig. 10b**)**. Liver showed severe vascular congestion with extravasated erythrocytes and perivascular mononuclear inflammatory cells infiltration of mainly lymphocytes **(**Fig. 10c**)**, diffuse vacuolation of hepatocytes of mainly fatty degeneration also appeared **(**Fig. 10d**)**. Most fish’ livers showed perivascular inflammatory mononuclear cells infiltration and severe vascular congestion **(**Fig. 10e**)** while spleen showed severe vascular congestion and focal extravasated erythrocytes (hemorrhage)** (**Fig. 10f**).** Focal fibrosis surrounded with inflammatory reaction represented in vascular dilation and inflammatory cells infiltration also appeared **(**Fig. 10g**)**. Intestine with moderate focal extravasated erythrocytes in the lamina propria, focal fibrosis of submucosa **(**Fig. 10h**)** while most cases showed diffuse congestion of submucosal blood vessels **(**Fig. 10i**).** Red tilapia subjected to P*aratapus undulatus* only (group 3) exhibited apparently normal gills with elongated primary lamellae, normal gill arch and normal straight secondary lamellae **(**Fig. 11a**)**. Liver showed apparently normal hepatic parenchyma and cellular details except in focal mild cellular vacuolation of mainly hydropic degeneration represented in swollen cells with central nucleus and vacuolated cytoplasm **(**Fig. 11b**)** while spleen showed normal parenchyma and mild subcapsular lymphocytic cellular depletion **(**Fig. 11c**)**. Intestine revealed normal mucosa, submucosa and serosa except in mild submucosal vascular congestion **(**Fig. 11d**)**.

**Fig. 12:**
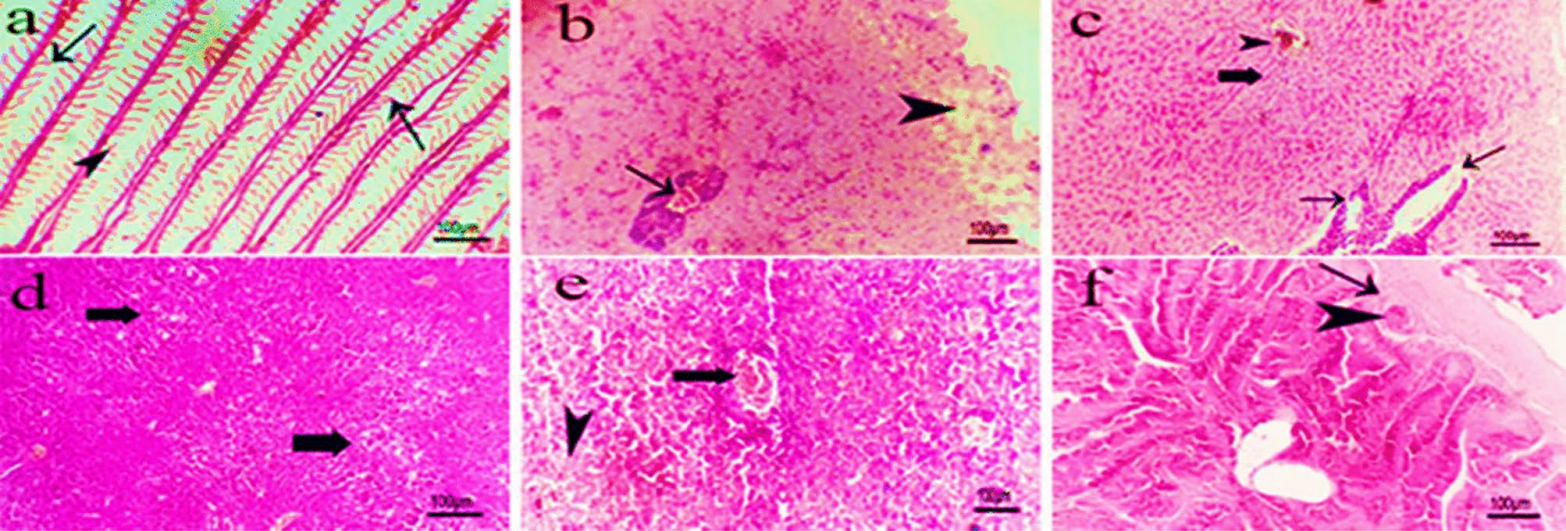
Photomicrograph of H&E section of different organs of red tilapia infected with *V. alginolyticus* and subjected to *P. undulatus* showing: **a)** gills with apparently normal primary lamellae, fusion of some secondary lamellae (arrows) and curving of others (arrow head). **b)** liver with mild vascular congestion (arrow) and focal mild subcapsular hepatocytes vacuolation (arrow head). **c)** liver with partial destruction of melano-macrophage centers (thin arrows), mild vascular congestion (thick arrow) and few mononuclear inflammatory cells infiltration (arrow head). **d)** spleen with well-organized parenchyma and mild focal lymphocytic cells depletion from white pulp (thick arrows). **e)** spleen with mild vascular congestion (thick arrow) and mild focal lymphocytic cells depletion from white pulp (arrow head). **f)** intestine with mild submucosal vascular congestion (arrow head) and perivascular mononuclear inflammatory cells infiltration (arrow head). (Scale bar = 100 µm).

Fish of group **(4)** infected with *V. alginolyticus* and subjected to *P. undulatus* showed apparently normal gills with intact primary lamellae, fusion of some secondary lamellae and curving of others **(**Fig. 12a**)**. were demonstrated while liver revealed mild vascular congestion and focal mild subcapsular hepatocytes vacuolation **(**Fig. 12b**)**. Partial destruction of melano-macrophage centers within hepatic parenchyma, mild vascular congestion and few mononuclear inflammatory cells infiltration were also detected **(**Fig. 12c**)** while the spleen showed well-organized parenchyma and mild focal lymphocytic cells depletion from white pulp **(**Fig. 12d**)**. Mild vascular congestion and mild focal lymphocytic cells depletion from white pulp **(**Fig. 12e) were also detected**.** Intestine showed mild submucosal vascular congestion and perivascular mononuclear inflammatory cell infiltration of mainly lymphocytes **(**Fig. 12f**)**.

Moreover, Table 5 presents the lesion scores for various pathological changes observed in the different experimental groups.

**Table 5:**
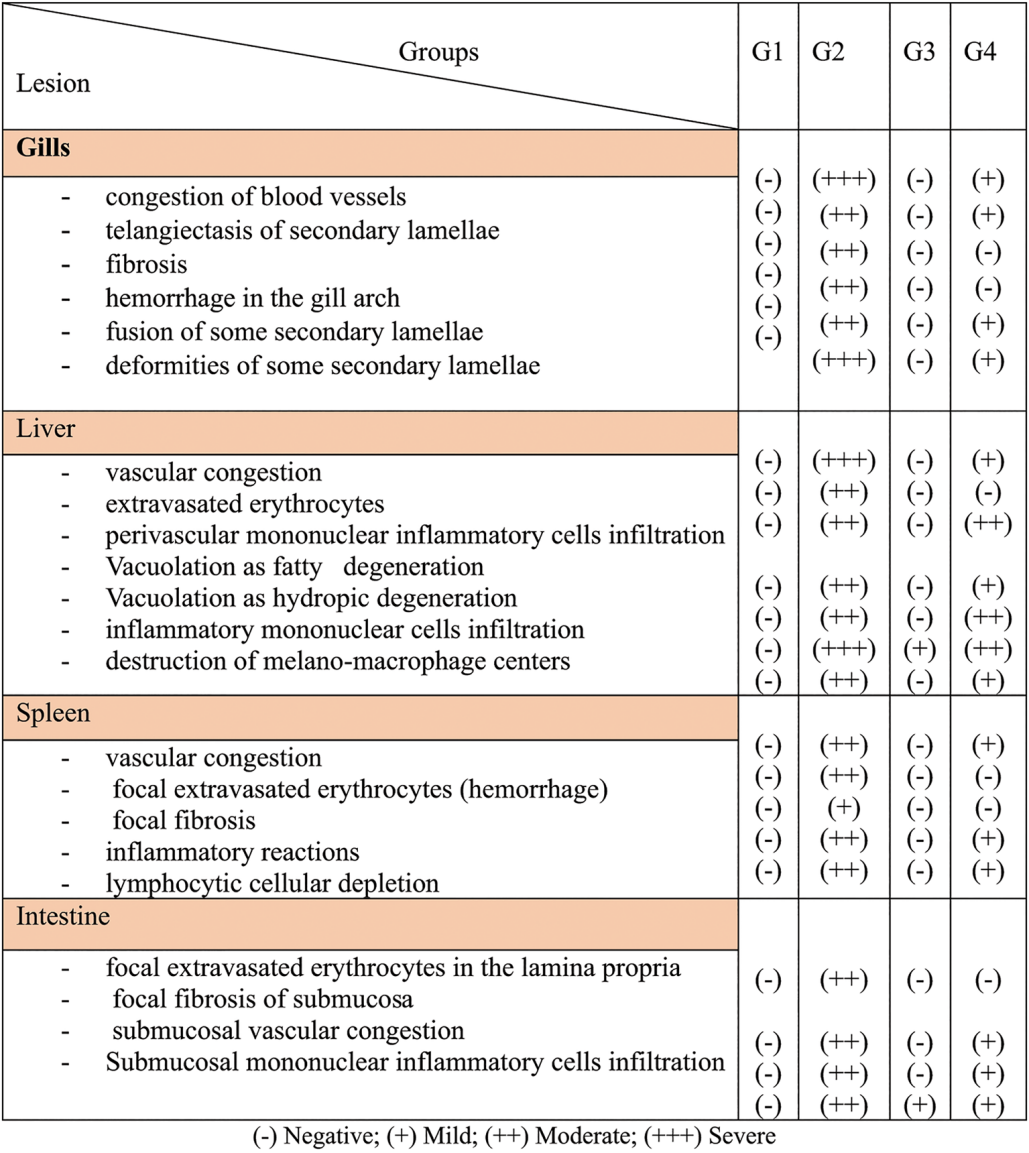
Lesion score of different pathological changes in different experimental groups.

(-) Negative; (+) Mild; (+ +) Moderate; (+ + +) Severe.

### Antimicrobial activity of P. undulata methanolic extract

**Fig. 13:**
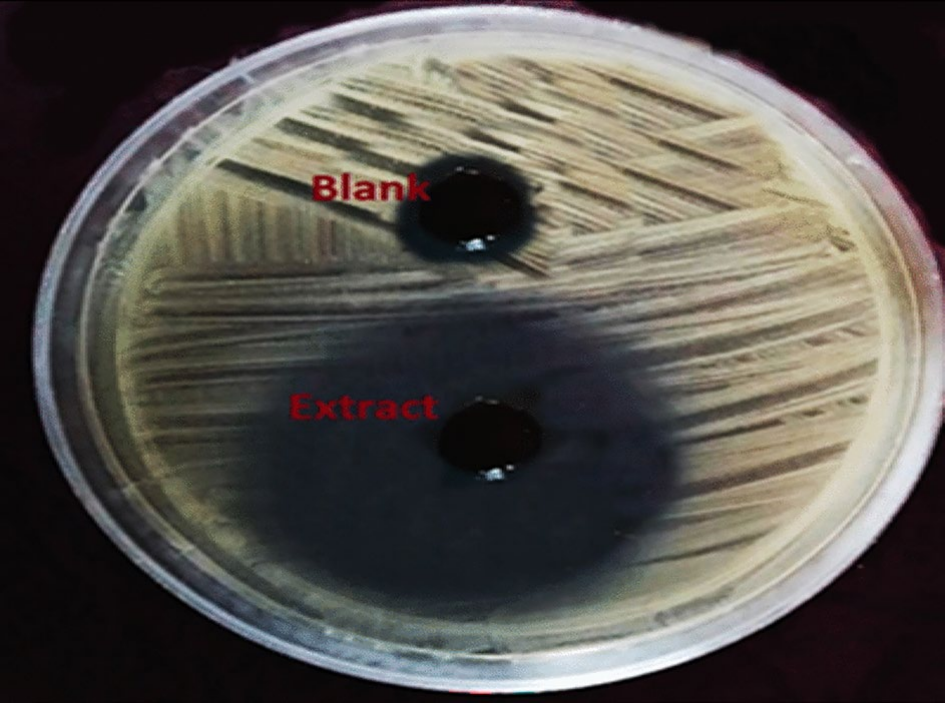
Agar well diffusion assay results of *P. undulata* Extract and blank on a lawn of pan-drug resistant *V. alginolyticus* isolate.

The *P. undulata* methanolic extract exhibited significant antimicrobial activity against the pan-drug resistant *V. alginolyticus* isolate. In vitro assays demonstrated a pronounced zone of inhibition of 30 mm (Fig. 13) in the agar well diffusion test. Further analysis revealed a potent minimum inhibitory concentration (MIC) of 4 µg/mL against the bacterium.

### Analysis of GC–MS Data

GC–MS analysis of the *P. undulata* extract revealed a diverse chemical profile, encompassing phenols, fatty acids and their esters, amides, and sterols. Notably, the extract was rich in natural products, particularly cholesterol derivatives such as desmosterol, a precursor to cholesterol with potential anti-inflammatory and anti-proliferative properties; stigmasterol, a plant sterol exhibiting anti-inflammatory, antioxidant, and anti-cancer activities; and ergosterol, a precursor to vitamin D with potential anti-microbial and anti-cancer properties (**Figure S5 and Table S2**). This diverse chemical composition suggests that the observed biological activities of *P. undulata* may arise from the synergistic effects of multiple compounds.

### FTIR analysis

FTIR analysis of the *P. undulata* extract revealed characteristic absorption bands, including 1656 cm⁻^1^ (C = O stretching of ester groups), 1650–1436 cm⁻^1^ (C = C stretching of aromatic rings), and 1147 cm⁻^1^ (C-O stretching of ether groups). These spectral features strongly suggest the presence of various functional groups, aligning with the GC–MS analysis, which identified phenols, esters, and other organic compounds within the extract.

## Discussion

Vibriosis, a significant global threat to aquaculture, is primarily caused by bacterial pathogens from the genus *Vibrio*, with *V. alginolyticus* being a major contributor. This bacterium poses a serious threat to tilapia aquaculture worldwide, causing significant economic losses due to high mortality rates, impaired growth, and reduced feed conversion efficiency^62^. *Vibrio alginolyticus* infections not only weaken the tilapia’s immune system, increasing susceptibility to secondary infections, but also pose a zoonotic risk, potentially affecting human health through the consumption of contaminated seafood^5,63^.

The prevalence of *V. alginolyticus* in our study (12%) from random collected fish samples in markets was comparable to previous reports^64,65^, but lower than recent studies^4,66,67^. These variations can be attributed to factors such as fish species, geographic location, and sampling season.

The presence of virulence genes, such as *tdh* and *trh*, is crucial for the pathogenicity of *V. alginolyticus*^68^. The *tdh* gene encodes a thermostable direct hemolysin, which can lyse red blood cells and damage host tissues^69^. The *trh* gene encodes a TDH-related hemolysin, which exhibits similar hemolytic activity^70^. The *toxR* gene, a transcriptional regulator, plays a pivotal role in controlling the expression of virulence genes, including *tdh* and *trh*^71^. The high prevalence of these virulence genes in our study suggests a high potential for these isolates to cause severe disease outbreaks.

Antibiotic susceptibility testing revealed a concerning prevalence of extreme drug-resistant (XDR) *V. alginolyticus* isolates, with 33.33% exhibiting resistance to multiple antibiotic classes, including ampicillin, doxycycline, and erythromycin. While some isolates remained sensitive to oxolinic acid and trimethoprim/sulfamethoxazole, and moderately sensitive to oxytetracycline, the emergence of these XDR strains underscores the urgent need for alternative therapeutic strategies. This high level of antibiotic resistance poses a significant public health threat, limiting treatment options for both human and animal infections and contributing to the global spread of antibiotic resistance^72–78^.

Despite the relatively low overall prevalence of *V. alginolyticus* in the total fish samples (2–3%), its significance in this study is justified by its dominance among *Vibrio* spp. isolates (12%), the high virulence gene presence (*tdh*, *trh*, *toxR*), and the substantial proportion of XDR strains. These characteristics signal a high potential for severe disease outbreaks. Moreover, *V. alginolyticus* is a known pathogen of red tilapia, the model species used in our challenge experiments, making it directly relevant to our research objectives. Ultimately, this study aimed to evaluate the therapeutic potential of *P. undulata* against a clinically relevant and challenging *Vibrio* pathogen, and the identified XDR *V. alginolyticus* isolates fulfilled this criterion.

Marine organisms, particularly bivalves, are a promising source of bioactive compounds with antimicrobial properties. *P. undulata* is a marine bivalve known to produce various antimicrobial peptides, making it a potential candidate for developing natural antimicrobial agents^11^.

To evaluate the in vivo efficacy of *P. undulata* against *V. alginolyticus*, we utilized a red tilapia infection model. Red tilapia is a well-established model for studying fish diseases due to its susceptibility to various pathogens and the similarity of its disease manifestations to natural infections^79^. By employing an intraperitoneal open culture system model, we were able to precisely control the infection dose and monitor the progression of the disease.

Clinical signs and gross lesions associated with vibriosis can vary depending on the specific *Vibrio* species, infection route, and fish species. In this study, fish infected with *V. alginolyticus* exhibited a range of clinical signs, including lethargy, anorexia, erratic swimming behavior, and skin lesions. Grossly, these fish displayed fin erosion, gill congestion, and internal organ damage, including hepatic and splenic lesions. These findings are consistent with previous studies^80–82^.

Interestingly, fish treated with *P. undulata* treatment exhibited significantly reduced clinical signs and gross lesions compared to the infected control group. This suggests that this system may have protective effects against *V. alginolyticus* infection. The reduced severity of clinical signs and lesions in the treated group may be attributed to the antimicrobial, antioxidant, and immunomodulatory properties of the treatment^83^, as evidenced by the following findings.

Hematological parameters serve as valuable indicators of fish health status. In the present study, the infected control group (G2) exhibited significant decreases in red blood cell (RBC) count, hemoglobin concentration, and packed cell volume (PCV), accompanied by leukocytosis, lymphocytosis, neutrophilia, and monocytosis. These alterations are consistent with previous findings reported in fish infected with *V. alginolyticus*^74,84^, suggesting a compromised immune response and impaired oxygen transport capacity.

Conversely, the infected and treated group (G4) demonstrated significant improvements in hematological parameters, including increased RBC count, hemoglobin concentration, and PCV, while exhibiting a reduction in white blood cell (WBC) count. These findings suggest that the supplementation with *P. undulata* mitigated the detrimental effects of *V. alginolyticus* infection on hematological parameters. The observed increase in RBC count and hemoglobin concentration indicates enhanced erythropoiesis and improved oxygen-carrying capacity^85^, crucial for maintaining vital physiological functions. Furthermore, the reduction in WBC count suggests a downregulation of the inflammatory response and a gradual return towards immune homeostasis^86^, indicating a positive influence of *P. undulata* on the fish’s immune system.

Our findings demonstrate that *P. undulata* supplementation effectively mitigated *V. alginolyticus*-induced hematological abnormalities in red tilapia. The observed improvements in RBC parameters, indicating enhanced oxygen transport, and the reduction in WBC count, suggesting a modulated inflammatory response, underscore *P. undulata’s* potential as a dietary supplement for improved fish health. These findings align with previous studies showing that marine bivalves can enhance hematological parameters in fish under stress^87,88^.

Significant alterations in biochemical parameters, including reduced liver and kidney enzyme levels, confirm *P. undulata’s* protective effects against *V. alginolyticus* infection. The increased total protein levels in treated groups, reflecting enhanced tissue repair, are likely attributable to the anti-inflammatory and immunomodulatory properties of *P. undulata*. The presence of bioactive compounds such as stigmasterol, β-sitosterol, and campesterol, known to play crucial roles in modulating inflammation through interactions with signaling pathways like NF-κB^87,89–92^.

The biochemical and molecular findings of this study provide strong evidence for the protective effects of *P. undulata* against *V. alginolyticus* infection.

*P. undulata* significantly enhanced antioxidant capacity, as evidenced by the upregulation of SOD and CAT. This is consistent with previous studies demonstrating the antioxidant potential of marine bivalves^93^. The observed downregulation of GST, while seemingly paradoxical, may reflect a context-dependent shift in antioxidant defense, possibly minimizing glutathione conjugation of specific metabolites^94,95^. Polyphenols and hydrolysates, identified in *P. undulata*, are potent free radical scavengers and likely contribute to its antioxidant activity^96–98^.

While *V. alginolyticus* infection elicited a robust immune response, evidenced by elevated lysozyme, nitric oxide, and IgM, *P. undulata* treatment effectively modulated this response, preventing excessive inflammation. This is supported by the significant downregulation of pro-inflammatory cytokines (*IL-1β, IL-6*) and upregulation of anti-inflammatory cytokines (*IL-10, IL-18*). These immunomodulatory effects are likely mediated by the synergistic action of various bioactive compounds in *P. undulata*, including sterols, fatty acids, and polysaccharides, which are known to influence cytokine production and immune cell function^99–103^.

The infected control group (G2) exhibited elevated levels of lysozyme, nitric oxide, and IgM, indicative of a robust immune response to *V. alginolyticus* infection. These findings align with previous studies demonstrating the activation of innate and humoral immune responses in fish challenged with this pathogen^104–107^.

Interestingly, the *P. undulata*-treated groups (G3 and G4) showed a significant decrease in these immune parameters. While a robust immune response is crucial for pathogen clearance, excessive inflammation can be detrimental. The observed downregulation in these parameters in the treated groups suggests that *P. undulata* may exert immunomodulatory effects, potentially by balancing the immune response and preventing excessive inflammation.

Furthermore, the infected control group exhibited significant elevations in pro-inflammatory cytokines *(IL-1β, IL-6, IL-22, IL-34,* and *TNF-α*), consistent with previous findings on *V. alginolyticus* infection^108,109^. However, the *P. undulata*-treated group demonstrated a significant downregulation of pro-inflammatory cytokines (*IL-1β* and *IL-6*) at both transcriptional and protein levels. This observation, coupled with the upregulation of anti-inflammatory cytokines (*IL-10* and *IL-18*) in the treated group, strongly suggests that *P. undulata* can modulate the immune response by suppressing excessive inflammation and promoting immune homeostasis. This finding is supported by previous studies demonstrating the anti-inflammatory properties of marine-derived compounds^99–101^**.**

Our findings suggest that *P. undulata* supplementation can effectively modulate the immune response in red tilapia challenged with *V. alginolyticus*. By downregulating pro-inflammatory cytokines and potentially balancing the immune response, *P. undulata* may contribute to improved disease resistance and overall fish health.

Histopathological examination of the infected group (G2) revealed significant tissue damage in the liver, spleen, and intestine, characterized by hepatic congestion, vacuolation, hemorrhage, fibrosis, and inflammatory cell infiltration. Gill tissues exhibited telangiectasis, deformity, and fusion of secondary lamellae, along with hemorrhagic arches and partial fibrosis. These findings are consistent with previous studies on *V. alginolyticus* infections in fish^82,110,111^.

In contrast, the *P. undulata*-treated groups showed marked improvements:

- **G3 (Clam-treated Control):** Tissues appeared normal with minimal inflammatory cell infiltration, likely attributable to the administered *P. undulata*.
- **G4 (Clam-treated Infected):** Significantly reduced tissue damage was observed, with minimal inflammation and cellular degeneration. This amelioration is likely due to the protective effects of *P. undulata*, as detailed in other sections of this study.

The observed amelioration of histopathological lesions in the treated groups aligns with the gene expression analysis. The downregulation of metabolic genes, such as *PACK* and *TFAM*, in the *P. undulata*-treated group suggests a shift in metabolic activity toward energy conservation and immune function^112,113^. *PACK*, a key enzyme in gluconeogenesis, plays a crucial role in maintaining blood glucose levels^114^. Its downregulation in the treated group may indicate a shift towards glycolysis, providing energy for immune cell activation and proliferation. Given that our study observed significant changes in hematological and biochemical parameters, including potential shifts in energy metabolism during infection and treatment, *PACK* was chosen to assess the impact of *P. undulata* on glucose regulation. *TFAM*, a mitochondrial transcription factor, regulates mitochondrial biogenesis and function^115–117^. Downregulation of *TFAM* may lead to decreased mitochondrial activity, which can reduce oxidative stress and energy consumption. As our study aimed to investigate the immunomodulatory and antioxidant effects of *P. undulata*, *TFAM* was selected to examine the potential modulation of mitochondrial activity and its implications for cellular stress responses.

This metabolic adaptation could be beneficial during periods of stress or infection, as observed in the reduced tissue damage in the treated groups. These findings align with previous studies that have demonstrated the metabolic effects of marine-derived compounds, such as the ability of certain marine algae to modulate metabolic pathways in fish, leading to improved growth and stress tolerance^118,119^.

It is important to acknowledge that while gene expression analysis provides valuable insights into the molecular mechanisms underlying the observed effects, it does not necessarily reflect protein-level changes. Gene expression is a complex process influenced by various factors, including transcriptional regulation, mRNA stability, and translational efficiency. Therefore, changes in mRNA levels may not always directly correlate with corresponding changes in protein abundance or activity. Future studies should consider incorporating protein-level analyses, such as Western blotting or enzyme activity assays, to validate the gene expression findings and provide a more comprehensive understanding of the molecular mechanisms involved.

Furthermore, the significant downregulation of virulence genes, including *tdh*, *trh*, and *toxR*, in *V. alginolyticus* treated with *P. undulata* suggests that this treatment may interfere with the expression of virulence factors, thereby reducing bacterial pathogenicity. This finding is consistent with previous studies that have demonstrated the ability of marine-derived compounds to inhibit bacterial virulence gene expression^15,120^. The downregulation of these genes may be due to several mechanisms, including inhibition of quorum sensing, interference with bacterial signaling pathways, or direct binding to DNA^121^. By reducing the expression of virulence genes, *P. undulata* extract may contribute to a reduction in bacterial virulence and pathogenicity, ultimately leading to a decrease in the severity of infection and improved fish health.

The GC–MS analysis of the *P. undulata* extract revealed the presence of various bioactive compounds, including fatty acids, steroids, and terpenoids. These compounds have been implicated in a range of biological activities, including antimicrobial, antioxidant, and anti-inflammatory properties^122,123^. The presence of sterols like stigmasterol, β-sitosterol, and campesterol in *P. undulata* may contribute to its anti-inflammatory effects^124^. Furthermore, FTIR analysis of the *P. undulata* extract revealed the presence of characteristic functional groups such as carbonyl, hydroxyl, and amine groups, suggesting the presence of biomolecules like polysaccharides, proteins, and lipids. This compositional profile aligns with the observed biological activities, as these biomolecules are known to possess antimicrobial, antioxidant, and anti-inflammatory properties^101,125^. For instance, fatty acids can act as anionic surfactants, exhibiting antifungal and antibacterial activities. Additionally, phenols can chelate iron, potentially limiting its availability to pathogens and enhancing the host’s defense mechanisms^126^. These findings suggest that the observed beneficial effects of *P. undulata* may be attributed to the synergistic action of its diverse bioactive components. However, despite these promising results, field validation and commercial feasibility studies are essential for practical application.

## Conclusion

This study definitively demonstrates the significant potential of *P. undulata* as a natural and sustainable solution for mitigating *Vibrio alginolyticus* infections in aquaculture. The observed reduction in mortality, coupled with enhanced fish health parameters in an open culture system, strongly supports its practical application. Furthermore, the effective modulation of the host’s immune response, characterized by reduced oxidative stress and inflammation, highlights its multifaceted protective capabilities.

The identification of bioactive compounds, including phenols, fatty acids, and sterols, provides critical insights into the underlying mechanisms of *P. undulata*’s efficacy. These compounds likely orchestrate a complex interplay of direct antimicrobial activity, immune enhancement, and oxidative stress reduction. This understanding paves the way for targeted applications and optimized formulations.

The implications of these findings extend beyond disease management. *P. undulata* offers a promising avenue for antibiotic-free aquaculture practices, addressing the growing concerns surrounding antibiotic resistance and environmental sustainability. By providing a natural alternative to traditional antibiotics, it contributes to healthier ecosystems and safer seafood production.

Future research should focus on refining extraction and purification processes to maximize the yield and potency of bioactive compounds. Investigating the synergistic effects of these compounds and developing cost-effective delivery methods for practical aquaculture applications are also crucial. Furthermore, exploring the long-term effects of *P. undulata* supplementation on fish growth, reproduction, and overall ecosystem health will be essential for its widespread adoption. Future studies should investigate the specific pathways modulated by the identified compounds. Additionally, evaluating the efficacy of *P. undulata* against other relevant pathogens in aquaculture is crucial.

## Supplementary Information

Supplementary Information.

## Data Availability

All data generated or analyzed during this study are included in this research article and its supplementary information files.

## Abbreviations

ALT: Alanine transaminase
AST: Aspartate transaminase
CAT: Catalase
GC–MS: Gas chromatography-mass spectrometry
FTIR: Fourier transform infrared spectroscopy
KBr: Potassium bromide
LDH: Lactate dehydrogenase
P.undulata: Paratapes undulata
SOD: Superoxide dismutase
