# Supplementary material for: Innovative vibriosis control in open aquaculture: Paratapes undulata as a sustainable growth and resistance enhancer in red tilapia
Source: Sci Rep. 2025 May 22;15:17750. doi: 10.1038/s41598-025-01026-x (PMC12098663; doi:10.1038/s41598-025-01026-x)
Supplement: Supplementary file 1 — Supplementary Information. [file 41598_2025_1026_MOESM1_ESM.docx]

**Table S1.** Antimicrobial Resistance pattern of *V. alginolyticus* isolates

| **Isolates** | **Resistance pattern** | **Resistance to antimicrobial groups** | **Resistance to antimicrobial agents** | **Resistance category** | **MARI** |
| --- | --- | --- | --- | --- | --- |
| 1 | OT- AM- FFC-DO -E | 4 | 5 | MDR | 0.71 |
| 2 | AM | 1 | 1 | SDR | 0.14 |
| 3 | AM- SXT-DO -E | 4 | 4 | MDR | 0.57 |
| 4 | OT- AM- FFC-DO -E | 4 | 5 | MDR | 0.71 |
| 5 | AM- FFC-DO -E | 4 | 4 | MDR | 0.57 |
| 6 | OT- AM- FFC-DO -E- OA | 5 | 6 | XDR | 0.85 |
| 7 | AM- FFC-DO -E | 4 | 4 | MDR | 0.57 |
| 8 | SXT - OT- AM- DO -E | 4 | 5 | MDR | 0.71 |
| 9 | SXT - AM- FFC-DO -E | 5 | 5 | XDR | 0.71 |
| 10 | SXT -OT- AM- FFC-DO -E | 5 | 5 | XDR | 0.85 |
| 11 | SXT-OT- AM- FFC-DO -E- OA | 7 | 7 | PDR | 1 |
| 12 | OT- AM- FFC-DO -E- OA | 5 | 6 | XDR | 0.85 |

E: Erythromycin; OT: Oxytetracycline; DO: Doxycycline; SXT: Trimethoprim/Sulfamethoxazole; AM: Ampicillin; OA: Qxolinic acid.

**Table S2. GC-MS Analysis of P. undulata Extract**

| Peak No. | Compound Name | Molecular Formula (Molecular Weight) | Area | Parent Ion (m/z) | Base Peak (m/z) |
| --- | --- | --- | --- | --- | --- |
| 1 | 2,4 Ditertiary butyl phenol (phenols) | C_14_H_22_O (206.0) | 0.65 | 206.0 | 191.0 |
| 2 | Ethyl g-hexadecencate (unsal. Fatty ester) | C_18_H_34_O_2_ (282.0) | 1.24 | 282.0 | 55.00 |
| 3 | Ethl palimitate (Sat. Fatty acid or ethyl hexadecencate) | C_18_H_36_O_2_ (284.0) | 0.76 | 284.0 | 88.00 |
| 4 | N-(2-Hydroxy ethyl octadecanamide or clindrol 200-Ms (Amide) | C_20_H_41_NO_2_ (327.0) | 0.62 | 327.0 | 85.00 |
| 5 | Ethyl 5,8,11,14,17- ico sapentaneoate (unsat. Fatty ester) | C_22_H_34_O_2_ (330.0) | 2.45 | 330.0 | 79.00 |
| 6 | 2,2- methylenebis(6 tertbutyl-p-cresol) (phenols) | C_23_H_32_O_2_ (340.0) | 0.80 | 340.0 | 177.0 |
| 7 | Methyl (47,72,107,137,167,197)-4,7,10,13,16,19- docosahexaenoate (unsal. Fatty ester) | C_23_H_34_O_2_ (342.0) | 0.63 | 342.0 | 79.00 |
| 8 | Bis (2 ethylhexyl phtholale (esters) | C_24_H_38_O_4_ (390.0) | 4.23 | 390.0 | 149.0 |
| 9 | 3-phenyl- (2E)-2- propeneic acid (acids) | C_9_H_8_O_2_ (148.0) | 7.63 | 148.0 | 103.0 |
| 10 | O-methyloscime- 17-Methoocyandrost-4-ea-3-one (Natural products) (cholesterol) | C_21_H_33_NO_2_ (331.0) | 0.71 | 331.0 | 125.0 |
| 11 | Tris (2,4- ditert-bulyl. Phenyl) phosphate (phosphrous cpds) | C_42_H_63_O_4_P (662.0) | 10.42 | 662.0 | 316.0 |
| 12 | Desmosterol (Natural products) (cholesterol) | C_27_H_44_O (384.0) | 8.91 | 384.0 | 69.00 |
| 13 | Cholesterol (Natural products) | C_27_H_46_O (386.0) | 26.06 | 386.0 | 43.00 |
| 14 | Vitamin E | C_29_H_50_O_2_ (430.0) | 1.23 | 430.0 | 43.00 |
| 15 | (3a^,^ ,22E , 24S) ergosta- 5,22- dien- 3-ol. (Natural products) | C_29_H_46_O (398.0) | 22.17 | 398.0 | 69.00 |
| 16 | Stigmasterol (Natural products) | C_29_H_48_O (412.0) | 5.07 | 412.0 | 55.00 |
| 17 | Sitosterol (Natural products) | C_29_H_50_O (414.0) | 3.33 | 414.0 | 43.00 |


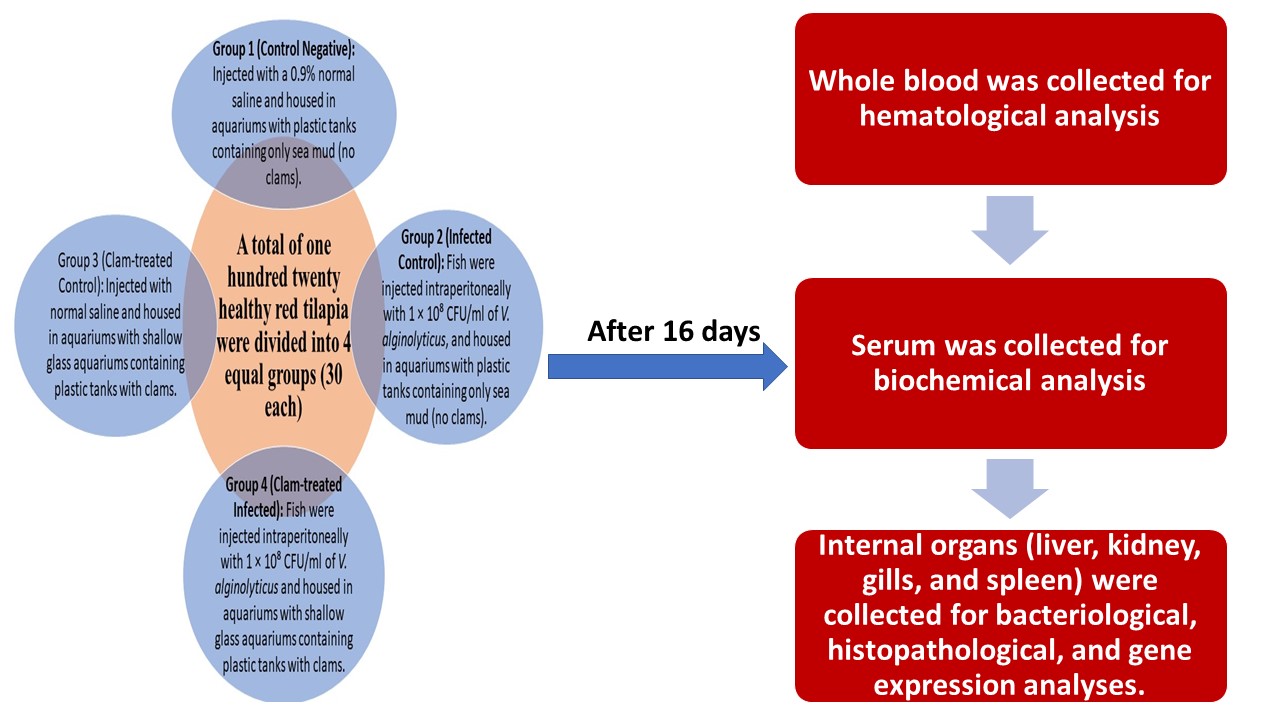


**Figure S1.** Experimental Design Flowchart


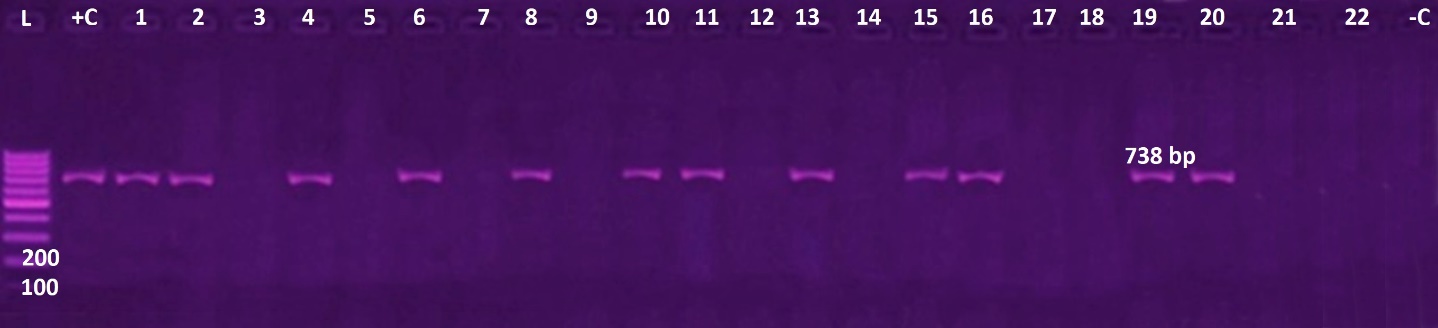


**Figure S2.** Conventional PCR amplification of the collagenase gene.


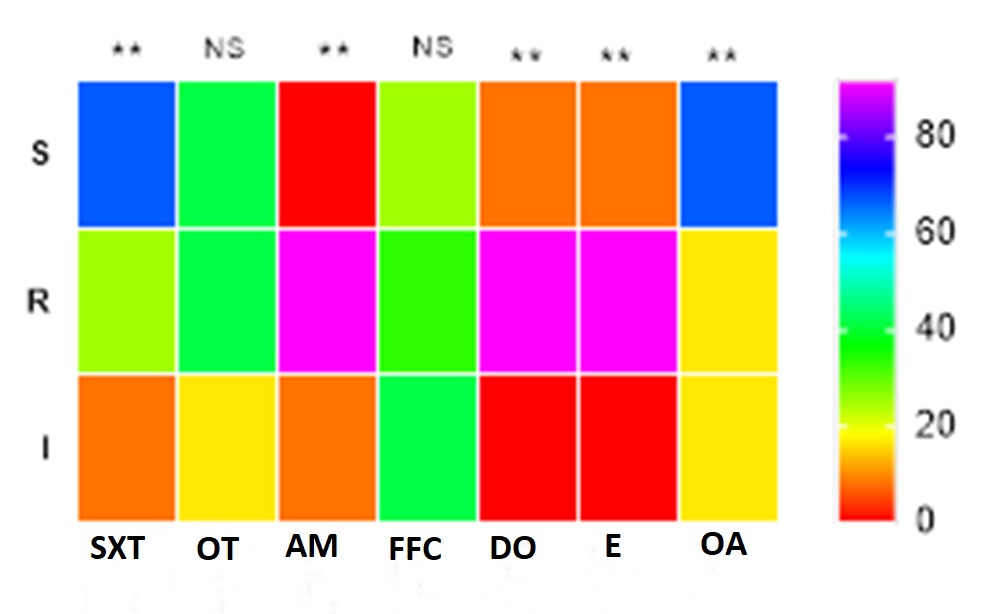


**Figure S3.** Antibiotic susceptibility profile of Vibrio alginolyticus isolates

**Figure S4.** The relationship between the presence of specific virulence genes (*trh, tdh,* and *toxR*) and antibiotic resistance patterns in V. alginolyticus isolates.


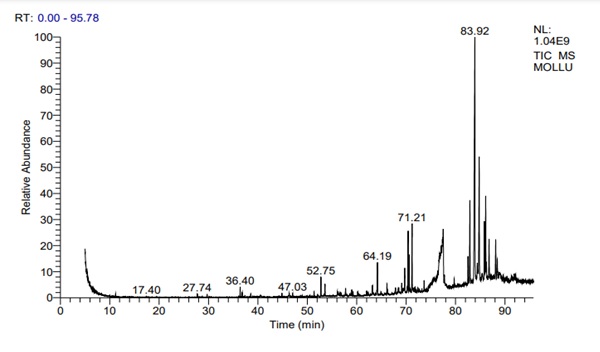


**Figure S5.** GC chromatography photo of *Paratapes undulata* methanolic extract
